# Extracellular vesicles: Natural liver‐accumulating drug delivery vehicles for the treatment of liver diseases

**DOI:** 10.1002/jev2.12030

**Published:** 2020-12-09

**Authors:** Gensheng Zhang, Xiaofang Huang, Huiqing Xiu, Yan Sun, Jiming Chen, Guoping Cheng, Zhengbo Song, Yanmei Peng, Yingying Shen, Jianli Wang, Zhijian Cai

**Affiliations:** ^1^ Department of Critical Care Medicine of the Second Affiliated Hospital Zhejiang University School of Medicine Hangzhou China; ^2^ Department of Comprehensive Medical Oncology Zhejiang Cancer Hospital Hangzhou China; ^3^ Institute of Immunology, and Department of Orthopedics of the Second Affiliated Hospital Zhejiang University School of Medicine Hangzhou China; ^4^ Department of Pathology Zhejiang Cancer Hospital Hangzhou China; ^5^ Department of Medical Oncology Zhejiang Cancer Hospital Hangzhou China; ^6^ Institute of Immunology, and Bone Marrow Transplantation Center of the First Affiliated Hospital Zhejiang University School of Medicine Hangzhou China; ^7^ Institute of Hematology Zhejiang University & Zhejiang Engineering Laboratory for Stem Cell and Immunotherapy Hangzhou China

**Keywords:** delivery vehicles, extracellular vesicles, liver accumulation, liver diseases, red blood cells

## Abstract

Extracellular vesicles (EVs) are excellent potential vectors for the delivery of therapeutic drugs. However, issues with biological safety and disease targeting substantially limit their clinical application. EVs from red blood cells (RBC‐EVs) are potential drug delivery vehicles because of their unique biological safety. Here, we demonstrated that EVs, including RBC‐EVs, show natural liver accumulation. Mechanistically, the liver environment induces macrophages to phagocytize RBC‐EVs in a C1q‐dependent manner. RBC‐EVs loaded with antisense oligonucleotides of microRNA‐155 showed macrophage‐dependent protective effects against acute liver failure (ALF) in a mouse model. These RBC‐EVs were also effective in treatment of ALF. Furthermore, compared to routine doses of doxorubicin and sorafenib (SRF), RBC‐EVs loaded with doxorubicin or SRF showed enhanced therapeutic effects on a murine model of orthotopic liver cancer through a mechanism dependent on macrophages. Importantly, drug‐loaded RBC‐EVs showed no systemic toxicity at therapeutically effective doses, whereas routine doses of doxorubicin and SRF showed obvious toxicity. Thus, drug‐loaded RBC‐EVs hold high potential for clinical applications in the treatment of liver disease therapy.

## 1 INTRODUCTION

The morbidity and mortality due to liver diseases have greatly increased over recent years worldwide (Li et al., 2020). According to different aetiologies, liver diseases can be categorized as viral hepatitis, acute liver failure (ALF), alcoholic or nonalcoholic liver diseases, cholestatic liver diseases, cholangiopathies, autoimmune liver diseases, cirrhosis and hepatic malignancies (Asrani, Devarbhavi, Eaton, & Kamath, 2019; Li et al., 2020). The treatment of liver diseases, especially ALF and hepatic malignancies, is still rather challenging. Because of the sudden loss of hepatic function, ALF has a high rate of mortality. The mortality rate was approximately 100% when ALF was first described nearly five decades ago. With the development of treatments, the mortality rate of ALF has substantially decreased. However, the overall mortality is still above 33% in the US (Riordan & Williams, 2008). Treatments for ALF are limited to orthotopic liver transplantation and the use of artificial livers in the clinic, both of which are highly invasive. MicroRNA‐155 (miR‐155) has been shown to aggravate liver injury (Liu et al., 2020; Zhang et al., 2019). Downregulation of miR‐155 by antisense oligonucleotides (ASOs) of miR‐155 (miR155‐ASOs) was reported to alleviate septic liver injury by inhibiting oxidative stress (Yang et al., 2018), suggesting these molecules are promising therapeutic drugs for ALF. However, efficient delivery of miR155‐ASOs to the liver is a major challenge. Liver cancer is the fourth leading cause of cancer‐related death worldwide (Bray et al., 2018). Despite advances in its treatment, liver cancer remains one of the most difficult cancers to treat (Siegel, Miller, & Jemal, 2020). Therefore, exploiting new strategies to treat liver diseases is urgent and important.

Extracellular vesicles (EVs) consist of ectosomes and exosomes. Ectosomes are vesicles generated by the direct outward budding of the plasma membrane including microvesicles, microparticles and large vesicles. Exosomes are released after the fusion of multivesicular bodies with the plasma membrane (Kalluri & Lebleu, 2020). EVs carrying proteins, lipids and RNAs from their parent cells play a crucial role in communication between cells (Mathieu, Martin‐Jaular, Lavieu, & Théry, 2019; O'brien, Breyne, Ughetto, Laurent, & Breakefield, 2020). EVs have been identified as potential delivery vehicles of RNA and chemotherapeutic reagents. Unlike viral gene transfer vectors, EVs are natural nanocarriers from endogenous cells with very low cytotoxicity and immunogenicity (Elsharkasy et al., 2020). EVs can protect the loaded RNAs from RNase and phagocytosis (Kamerkar et al., 2017; Usman et al., ). By avoiding the endosomal pathway, EVs can strongly enhance the transfection efficiency of siRNAs (Orefice, 2020). EVs can also decrease the accumulation of chemotherapeutic drugs in nontarget organs, thus decreasing the off‐target toxicity. In addition, the therapeutic effects of drugs are augmented when EVs are used (Cabeza et al., 2020; Xue, Wong, Song, & Cho, 2020; Zocchi, Tosetti, Benelli, & Poggi, 2020). For example, EVs carrying oncogenic *KRAS* siRNA suppressed cancer in multiple mouse models of pancreatic cancer and significantly increased overall survival (Kamerkar et al., 2017). EVs loaded with miR‐214‐ASOs could reverse cisplatin resistance in gastric cancer (Wang et al., 2018). EVs loaded with paclitaxel exhibited potent therapeutic effects against murine lung carcinoma (Kim et al., 2016). EVs encapsulating doxorubicin (Dox) greatly inhibited breast tumour growth without overt toxicity (Tian et al., 2014). EVs have also been reported to effectively prevent liver fibrosis by delivering miR‐181‐5p (Qu et al., 2017). Red blood cell extracellular vesicles (RBC‐EVs) have also been reported to deliver RNA drugs with high efficacy. RBC‐EVs loaded with miR125b‐ASOs notably suppressed the growth of human breast cancer and showed excellent therapeutic effects on xenograft mouse models of human acute myeloid leukaemia (Usman et al., 2018). Given their high production and beneficial characteristics in biosafety, RBC‐EVs are probably the potential delivery vectors for therapeutic drugs for liver diseases.

An increase in the EV‐specific targeting of diseases can largely enhance the delivery efficacy of drugs and optimize the therapeutic effects for the corresponding diseases. EVs incorporating aminoethylanisamide‐polyethylene glycol targeted lung cancer and possessed improved antitumor effects after encapsulating paclitaxel (Kim et al., 2018). EVs displaying single chain variable fragments were efficiently targeted to tumour cells expressing a cognate antigen (Longatti et al., 2018). EVs containing membrane integrin αv‐specific iRGD peptides showed highly efficient targeting and Dox delivery to integrin αv‐positive breast cancer cells, leading to improved antitumor effects (Tian et al., 2014). However, integrin signalling can activate pro‐inflammatory *S100* gene expression, which is correlated with tumour metastasis (Hoshino et al., 2015). EVs from CD63 and a sequence from Apo‐A1 fusion gene‐modified 293T cells selectively bound to HepG2 liver cancer cells via the scavenger receptor class B type 1‐Apo‐A1 complex. After loading with miR‐26a, these EVs showed enhanced suppressive effects in HepG2 cells (Liang et al., 2018). Whether there are undesirable side effects due to receptor internalization after scavenger receptor class B type 1‐Apo‐A1 binding is unknown. Similarly, rabies viral glycoprotein‐derived peptide, which can bind to its specific receptor (acetylcholine receptor), is used to target EVs to the brain (Alvarez‐Erviti et al., 2011). Whether the binding of rabies viral glycoprotein‐derived peptide will activate the acetylcholine receptor and the effects mediated by the activated receptor are unknown. Therefore, although artificially modified cell‐targeting EVs show strong drug delivery efficacy, they potentially increase the risks of clinical applications involving EVs. Whether there are EVs that show with accumulation in some cells or organs that would make them the optimal candidates for drug delivery to these cells or organs has yet to be determined.

Here, we showed that RBC‐EVs naturally accumulate in the liver after intravenous injection in a predominantly macrophage‐dependent mechanism. The depletion of macrophages resulted in the redistribution and decreased liver accumulation of RBC‐EVs. RBC‐EVs loaded with miR‐155‐ASOs showed excellent protective and therapeutic effects against ALF. RBC‐EVs loaded with Dox or sorafenib (SRF) showed notable therapeutic effects against orthotopic liver cancer. Consistent with the redistribution of RBC‐EVs, the depletion of macrophages substantially blunted the therapeutic effects of the drug‐loaded RBC‐EVs, indicating the importance of the liver accumulation of RBC‐EVs for the treatment effect. These observations suggest that drug‐loaded RBC‐EVs are natural liver‐accumulating reagents that could be used for the treatment of liver diseases.

## 2 MATERIALS AND METHODS

### 2.1 Mice and human samples

C57BL/6J, BALB/c and BALB/c‐nude male mice aged 7–8 weeks were purchased from Joint Ventures Sipper BK Experimental Animal Co. (Shanghai, China). The mice were housed in a specific pathogen‐free facility, and the experimental protocols were approved by the Animal Care and Use Committee of the Zhejiang Cancer Hospital (2020‐05‐065). Human blood was obtained from 20 healthy donors with type O blood (75 ml per donor). The blood was collected in 10 ml purple‐top EDTA anticoagulant tubes at room temperature (RT). Normal lung, liver and kidney tissues were obtained from tumour patients. The characteristics of the patients are listed in Table S1. The fresh tissues were immediately placed in RPMI‐1640 (Basalmedia, Shanghai, China) containing 10% fetal bovine serum (FBS) (Thermo Fisher Scientific, Waltham, CA, USA) and 1% penicillin/streptomycin at 37°C in a humidified atmosphere with 5% CO_2_. The collection of human samples was approved by the local Ethical Committee and the Review Board of Zhejiang University (2019‐084) and Zhejiang Cancer Hospital (2020‐05‐065). All participants were informed of the usage of their samples, and informed consent was obtained.

### 2.2 Cell lines and cell culture

Murine RAW264.7 macrophages (TCM13), Hepa1‐6 hepatoma cells (SCSP‐52), 4T1 breast cancer cells (TCM32), human HepG2 hepatoma cells (SCSP‐50) and acute monocytic leukaemia THP‐1 cells (TCHu57) were purchased from the Type Culture Collection of the Chinese Academy of Sciences (Shanghai, China). Human HCC‐LM3 hepatoma was provided by Professor Weimin Fan (Zhejiang University, Hangzhou, China). Luciferase‐expressing B16F10 (B16F10‐Luci) and luciferase‐expressing HCC‐LM3 (HCC‐LM3‐Luci) cells were established in our laboratory. Briefly, pLVX‐EGFP‐luciferase plasmids were packaged into lentiviral particles using 293T cells cotransfected with the viral packaging plasmids psPAX2 and VSVG. Lentiviral supernatants were harvested 48 h after transfection. B16F10 and HCC‐LM3 cells were infected with filtered lentivirus supernatants for 4 h. Forty‐eight hours later, both cells were sorted into 96‐well plates by EGFP expression. Single cell clones were selected and expanded. B16F10‐Luci and HCC‐LM3‐Luci clones were identified by addition of luciferin and measurement with GloMax2020 (Promega, Madison, WI, USA). 4T1 cells were cultured in RPMI‐1640 supplemented with 10% exosome‐depleted FBS (Thermo Fisher Scientific) and 1% penicillin/streptomycin. THP‐1 cells or other cells were cultured in RPMI‐1640 or DMEM (Basalmedia) supplemented with 10% FBS and 1% penicillin/streptomycin, respectively. For the generation of dendritic cells (DCs), bone marrow mononuclear cells were prepared from C57BL/6J mouse (6–8 weeks old) tibial and femur suspensions by depletion of red blood cells and cultured at a density of 2 × 10^6^ cells/ml in 6‐well plates in RPMI‐1640 medium supplemented with 10% exosome‐depleted FBS (Thermo Fisher Scientific), 1% penicillin/streptomycin, 10 ng/ml recombinant murine GM‐CSF and 1 ng/ml mouse IL‐4 (Millipore, Billerica, MA, USA). Nonadherent cells were gently washed out after 48 h of culture; the remaining loosely adherent clusters were cultured for another 48 h, and the supernatant was harvested for subsequent experiments. Human umbilical vein endothelial cells (HUVECs) were isolated as previously described (Baudin, Bruneel, Bosselut, & Vaubourdolle, 2007). Briefly, the clean vein of the umbilical cord was lavaged with 0.2% (w/v) collagenase solution at 37°C for 10 min. Then, the cells were collected, centrifuged, resuspended in complete M199 medium (Corning Incorporated, New York, CA, USA) and seeded in 35 mm sterile Petri dishes (passage 1). HUVECs (passages 2–6) were cultured in M199 (53%)/human endothelial serum‐free mixed medium (37%) with 10% FBS (Thermo Fisher Scientific) and endothelial cell growth supplement (15 μg/ml, Sigma‐Aldrich, St. Louis, MO, USA). All cells were cultured at 37°C with 5% CO_2_. THP‐1 cells were differentiated for 48 h with 50 ng/ml phorbol myristate (Sigma‐Aldrich). Peritoneal macrophages (PMs) were collected 4 days after thioglycollate (Millipore) injection.

### 2.3 Separation of extracellular vesicles

The red blood cell extracellular vesicles (RBC‐EVs) were separated as previously described (Usman et al., 2018). Briefly, RBCs were separated from plasma and white blood cells by centrifugation at 500 × *g* for 10 min and passed through a leukodepletion filter (Terumo, Tokyo, Japan). Isolated RBCs were diluted in PBS and treated with 1 μM calcium ionophore (Sigma‐Aldrich) overnight at 37°C. Then, the RBCs and cell debris were removed by centrifugation at 600 × *g* for 20 min, 1600 × *g* for 15 min, 3260 × *g* for 15 min, and 10,000 × *g* for 30 min at 4°C. For 4T1‐EV and DC‐EV separation, cell culture supernatants were centrifuged at 500 × *g* for 10 min, 2000 × *g* for 15 min and 10,000 × *g* for 30 min at 4℃. Then, the supernatants were passed through 0.22 μm syringe filters (Millipore) and collected in 35 ml ultracentrifuge tubes (#344058, Beckman Coulter, Brea, CA, USA). The EVs were concentrated using ultracentrifugation with a SW32Ti rotor (L‐90K with SW32Ti rotor, Beckman Coulter) at 100,000 × *g* for 70 min at 4°C. Subsequently, the EV pellets were resuspended in sterile PBS. For further concentration of the EVs, the pellets were resuspended in 0.25 M sucrose and floated into a discontinuous density cushion composed of 25% sucrose/45% sucrose (pH 7.2) (at the expected density of EVs of 1.11–1.21 g/ml (Valadi et al., 2007)) for 18 h at 100,000 × *g* with a SW32Ti rotor. The protein contents of the EVs were quantified by using a BCA protein assay kit in the absence of detergent (Thermo Fisher Scientific). We have submitted all relevant data of our experiments to the EV‐TRACK knowledgebase (EV‐TRACK ID: EV200089) (Consortium et al., 2017).

### 2.4 Western blotting

A total of 10 μg of RBC‐EV, cell or tissue lysates was resuspended in 5 × SDS loading buffer, subsequently incubated at 100°C for 5 min and centrifuged at 12,000 × *g* for 10 min. Then, the supernatants were separated by 10% SDS‐polyacrylamide gel (Thermo Fisher Scientific) electrophoresis and transferred to PVDF membranes (Millipore), which were blocked with 5% skim powdered milk (w/v) for 1.5 h, incubated with the relevant primary antibodies at 4°C overnight, and then incubated with secondary antibodies at RT for 2 h. An ECL Kit (MultiSciences, Hangzhou, Zhejiang, China) was used to detect the bands. The antibodies used and the corresponding dilutions are listed in Table S2.

### 2.5 Transmission electron microscopy

A total of 5 μg of RBC‐EVs was diluted in PBS and placed on 200‐mesh carbon‐coated copper grids at RT for 2 min. The excess suspension was removed using filter paper. Then, the RBC‐EVs were negatively stained with uranyl acetate at RT for 5 min, washed twice with PBS, dried and examined under an FEI Tecnai T10 electron microscope (FEI, Hillsboro, OR, USA) operating at 100 kV.

### 2.6 Nanoparticle tracking analysis

The number and size distribution of RBC‐EVs were analysed using the NanoSight NS500 (Malvern, Malvern, Worcestershire, UK). For recordings, samples were pumped automatically into a chamber at a constant flow rate using the Malvern NanoSight syringe pump system. The camera level was adjusted to 11, and three 60′ captures per sample were recorded. For analysis of the recordings, the detection threshold was set to 5 and the NTA3.3 Suite Software was used for analysis.

### 2.7 EV labelling

EVs were labelled using VivoTrack 680 (Fluorescence, Beijing, China), PKH26 (Sigma‐Aldrich) or carboxyfluorescein succinimidyl ester (CFSE, Thermo Fisher Scientific) according to the manufacturer's instructions. For VivoTrack 680 labelling, 150 μg EVs (150 μg ≈ 3.45 × 10^10^ particles) in 200 μl PBS were mixed with 42 μM VivoTrack 680 at RT for 30 min. For PKH26 labelling, 150 μg EVs were resuspended in 100 μl diluent C, 0.4 μl PKH26 ethanolic dye solution was added into another 100 μl diluent C. Then, 100 μl EV suspension was mixed with the 100 μl dye solution for another 5 min. For CFSE labelling, 150 μg EVs in 200 μl PBS were incubated with 7.5 μM CFSE at 37°C for 30 min. The staining was stopped by adding an equal volume of exosome‐depleted FBS (Thermo Fisher Scientific) and incubating for another 1 min. Finally, all the unbound dye was removed by ultracentrifugation at 120,000 × *g* for 90 min, and the pellets were resuspended in 200 μl PBS.

### 2.8 EV tracking

The labelled EVs (100 μg ≈ 2.3 × 10^10^ particles) were injected into mice via the tail vein or by intrathoracic or intraperitoneal injection. Twelve or twenty‐four hours later, the mice were sacrificed, and the organs of the brain, heart, lungs, liver, spleen, kidneys and gut were collected. For uptake of RBC‐EVs by human tissues, the lung, liver and kidney tissues were cut into small pieces with similar volumes and then cultured with 10 μg/ml VivoTrack 680‐labelled RBC‐EVs for 24 h at 37°C, followed by washing with PBS three times. Uptake of VivoTrack 680‐labelled EVs was imaged by an in vivo imaging system (IVIS, PerkinElmer, Waltham, MA, USA). Regions of interest were drawn to cover the entire organs. The background and autofluorescence were defined according to the supernatant negative controls and subtracted from the images using the Image‐Math function. PKH26‐ or CFSE‐labelled EVs were imaged by a Nikon A1R confocal microscope (Nikon, Tokyo, Japan). The filters for the IVIS systems were as follows: excitation filter 640–675 nm, barrier filter 680–780 nm. The filters for the Nikon systems were as follows: excitation filter 465–495 nm, barrier filter 512–558 nm to visualize CFSE; excitation filter 540–580 nm, barrier filter 600–660 nm to localize PKH26, and excitation filter 361–389 nm, barrier filter 430–490 nm to localize DAPI. Images were captured with a CCD camera (Orca I, Hamamatsu, Japan). VivoTrack 680 and PKH26 or CFSE tissue fluorescence images were acquired and analysed by Living Image version 4.4 software (Caliper Life Sciences, Hopkinton, MA, USA) and NIS‐Elements Viewer 4.2.0 (Nikon), respectively. To analyse the uptake of VivoTrack 680‐labelled EVs, three images were randomly selected from each photograph, and the radiant efficiency of the regions of interest in different organs was measured and recorded. For analysis of the uptake of PKH26‐ or CFSE‐labelled EVs, three images were randomly captured from each section. PKH26^+^ or CFSE^+^ cells in each field were counted. For the detection of luciferase expression in the orthotopic liver cancer and lung cancer model, the mice were intraperitoneally injected with 100 mg/kg luciferin 15 min before imaging, anesthetized with isoflurane, and imaged by an IVIS system. For the tumour burden analyses, Living Image version 4.4 software (Caliper Life Sciences) was used to quantify all tumours. A circular region of interest around the liver tumour was set within all experimental groups. In addition, the exposure conditions (time, aperture, stage position, and binning) were identical for all measurements within each experiment. The tumour measurements of total flux were obtained under the same conditions for all experimental groups.

### 2.9 Animal studies

To establish ALF model, mice were intraperitoneally injected with 100 μg/kg *Escherichia coli* 0111:B lipopolysaccharide (LPS, Sigma‐Aldrich) and 400 mg/kg d‐galactosamine (D‐GaIN, Sigma‐Aldrich) on day 0. To inhibition of the liver C1q levels in vivo, mice were intraperitoneally injected with 3,4‐dehydro‐l‐proline (DHP, 250 mg/kg) and sacrificed 24 h later. The sepsis model was induced by intraperitoneal injection of 500 μg/kg LPS on day 0. For determination of the protective effects on ALF and sepsis, mice were intravenously injected with antisense oligonucleotides of microRNA‐155 (miR‐155‐ASOs, 4 μg), RBC‐EVs loaded with miR‐negative control (NC)‐ASOs or miR‐155‐ASOs (100 μg ≈ 2.3 × 10^10^ particles) or 4 μg *N*‐acetylagalactosamine (GalNAc, Quanyang, Shanghai, China) on days ‐3, ‐2 and ‐1. The mice were sacrificed 24 h after treatment. In the therapeutic experiments for ALF, mice were injected with 50 μg/kg LPS and 200 mg/kg D‐GaIN on day 0. For investigation of the therapeutic effects on ALF, the mice were intravenously injected with miR‐155‐ASOs (4 μg), or RBC‐EVs loaded with miR‐NC‐ASOs or miR‐155‐ASOs (100 μg ≈ 2.3 × 10^10^ particles) 6 h later and sacrificed 12 h after treatment. For the orthotopic liver cancer model, nude mice were subcutaneously injected with 1 × 10^6^ HCC‐LM3‐luci cells. When the tumours reached 100 mm^3^, they were collected and cut into 1 mm^3^ pieces. Then, the tumour pieces were adhered to the livers of anaesthetized nude mice after opening of the abdomen with biomedical glue (BaiYun, Guangzhou, Guangdong, China) on day 0. On day 7, the tumours were observed by IVIS, and the mice received intravenous injections with doxorubicin (Dox) (3 μg or 5 mg/kg), RBC‐EVs or RBC‐EVs loaded with Dox (100 μg ≈ 2.3 × 10^10^ particles), SRF (15 μg or 30 mg/kg) or RBC‐EVs loaded with SRF (100 μg ≈ 2.3 × 10^10^ particles) every 3 days. The mice were regularly imaged by IVIS and were sacrificed on day 27. For the B16F10 lung metastasis model, mice were intravenously injected with 1 × 10^6^ B16F10‐Luci cells on day 0. Then, the mice were intravenously injected with 100 μg RBC‐EVs/Dox every 3 days starting on day 7, and tumour progression was monitored by an IVIS system. The mice were sacrificed on day 21. In some experiments, macrophages were depleted by pretreatment with clodronate liposomes (Clodrosome) as previously reported (Piao et al., 2017). In the ALF prophylaxis experiments, mice were intravenously injected with 50 μl of Clodrosomes (SKU: CP‐005‐005, Clodrosome, Brentwood, TN, USA) on days ‐4, ‐3, ‐2 and ‐1. In the tumour experiments, mice were intravenously injected with 50 μl of Clodrosomes 12 h before each RBC‐EVs/Dox injection.

### 2.10 Immunofluorescence

Tissues were embedded in Tissue‐Tek Cryo‐O.C.T (Thermo Fisher Scientific) and processed to obtain 5 μm sections. Then, the tissue sections were stained with primary antibodies at 4°C overnight, followed by staining with the corresponding fluorescence‐labelled secondary antibodies at 4°C for 1 h. Finally, the nuclei were stained with 0.5 μg/ml DAPI for 20 min at RT. The stained sections were observed with a Nikon A1R confocal microscope (Nikon). The CD31^+^ cell area was analysed by the vessel analysis plugin in ImageJ (NIH, Bethesda, MD, USA). The percent of CD31^+^ cell area was determined and normalized to the area of the total cells. The antibodies used and the corresponding dilutions are listed in Table S2.

### 2.11 Isolation of Kupffer cells and F4/80^+^, F4/80^–^ cells

For generation of liver single cells, liver tissues were dissociated using a gentleMACS Dissociator (Miltenyi Biotec, Bergisch Gladbach, Germany) in C tubes (Miltenyi Biotec) with liver dissociation kit (Miltenyi Biotec) according to the preset program. For isolation of Kupffer cells (KCs), the cells were washed twice with RPMI‐1640 via centrifugation at 500 × *g* for 8 min at 4°C. Then, the pellet was resuspended in 50% Percoll and layered with 25% Percoll on the mixture, followed by centrifugation at 800 × *g* for 20 min. The KC‐enriched interface between 25% and 50% Percoll was aspirated and washed twice with RPMI‐1640. For isolation of F4/80^+^ and F4/80^–^ cells, the cells were incubated with 10 μl/ml FcR blocking antibody (STEMCELL, Vancouver, BC, V6A 1B6, Canada) and 6 μg/ml biotin‐conjugated anti‐F4/80 (Biolegend, San Diego, CA, USA) for 15 min at RT and subsequently mixed with 100 μl/ml biotin‐selection cocktail (STEMCELL) for 15 min at RT. The F4/80^+^ and F4/80^–^ cells were isolated using a magnet. The beads and bead‐free supernatants containing F4/80^+^ and F4/80^–^ cells, respectively, were collected, and washed twice with RPMI 1640.

### 2.12 Flow cytometric analysis

EVs were captured by 4 μm‐diameter aldehyde/sulfate latex beads (Thermo Fisher Scientific) as previously described (Jiang et al., 2016). Briefly, 20 μg EVs were incubated with 5 μl of beads for 15 min at RT in PBS, with a 20 μl final volume. The mixture was then transferred to 1 ml of PBS with gentle shaking for 1 h. After centrifugation, the pellet was blocked by incubation with 20 μl of exosome‐depleted FBS for 30 min. EV‐coated beads were washed thrice in PBS and resuspended in 50 μl of PBS. Cells or EV‐coated beads were washed in PBS with 1% BSA and collected by centrifugation at 400 × *g* or 3800 × *g* for 5 min at 4°C, respectively. Then, the cells or beads were incubated with the corresponding fluorescence‐labelled antibodies in 100 μl of PBS for 20 min at RT. After three washes in PBS, cells or beads were analysed by flow cytometry (Novocyte flow cytometer, Agilent Biosciences, San Diego, CA, USA), and the data were analysed using FlowJo software (TreeStar, Ashland, OR, USA). The cells or beads were initially gated based on FSC‐A and SSC‐A to exclude debris and dead cells and then gated based on FSC‐A and FSC‐H to exclude doublets and aggregates. The fluorescent‐positive cells or beads were gated in the appropriate fluorescent channels. The antibodies used are listed in Table S2.

### 2.13 RNA extraction and real‐time PCR analysis

Total RNA was isolated from tissues or cells with TRIzol (Thermo Fisher Scientific). Reverse transcription was performed with a Reverse Transcription Kit (Toyobo, Osaka, Japan). For reverse transcription of mature miRNAs, specific primers of miR‐155 and *U6* (GenePharma, Shanghai, China) were used; for reverse transcription of mRNAs, primers in Reverse Transcription Kit (Toyobo) were used. PCR analysis was performed with Power SYBR Green (TaKaRa, Dalian, Liaoning, China) by using the following reaction conditions: 95℃ for 30′ followed by 39 cycles of 95℃ for 5′ and 60℃ for 30′. For detection of miR‐155 expression, *U6* snRNA served as an internal control. For mRNA detection, *Actb* served as an internal control. The relative fold changes in the expression of miRNAs or mRNAs were calculated using the following equation: relative quantification  =  2^–ΔΔCT^. The primers used are listed in Table S3.

### 2.14 Lysates of tissues or cells

Cells were collected and centrifuged at 400 × g for 5 min, and the pellets were resuspended in RPMI‐1640 with 1% phenylmethylsulfonyl fluoride (Cell Signaling Technology, MA, USA). Lung, liver and kidney tissues obtained from mice or patients were cut into smaller pieces by scissors and then lysed in RPMI‐1640 with 1% phenylmethylsulfonyl fluoride (Cell Signaling Technology) using an ultrasound (Bioruptor Pico, Diagenode, Liège, Belgium). For cells, the ultrasound program was performed as follows: 20′ on, 30′ off for 30 cycles in a cold bath. For tissues, the ultrasound program was performed as follows: 30′ on, 40′ off for 40 cycles in a cold bath. The lysates were centrifuged at 12,000 × g for 10 min at 4℃ and the supernatant was collected. The concentrations of the lysates were measured by BCA protein assays (Thermo Fisher Scientific) and immediately used in further experiments. For stimulation, 3 × 10^5^ cells/ml were plated into 24‐well plates overnight, and then the cells were primed with tissue or cell lysates (100 μg/ml) or 10 μg/ml C1q (Hycult Biotech, Ouden, Netherlands) for 24 h and incubated for another 6 h in the presence of RBC‐EVs (20 μg/ml). Then, the cells were collected for flow cytometric analysis. For inhibition of C1q synthesis, 2.5 mM DHP (Sigma‐Aldrich) was added to Hepa1‐6, HepG2 or HCC‐LM3 cells for 24 h.

### 2.15 Transfection of siRNA

Hepa1‐6 cells were plated in 12‐well plates (4 × 10^5^ cells/well) and then transfected with 100 nM NC or *C1qa* siRNA for 48 h using Interferin siRNA Transfection reagent (Polyplus, Beijing, China) according to the manufacturer's instructions. Then, the effects of gene silencing were measured by western blotting. The siRNAs were synthesized by GenePharma, and the sequences are listed in Table S3.

### 2.16 Immunohistochemistry

Murine and human lung, liver and kidney tissue sections were routinely deparaffinized and rehydrated, followed by antigen retrieval using 10 mM sodium citrate buffer (pH 6.0). After blocking with 5% BSA, the slides were incubated with primary antibodies at 4°C overnight. Then, the sections were incubated with the corresponding HRP‐conjugated secondary antibodies at RT for 30 min. After washing three times with PBS, the sections were detected using 3,3′‐diaminobenzidine tetrahydrochloride (DAKO, Copenhagen, Denmark) and counterstained with haematoxylin according to the manufacturer's instructions. The antibodies used and the corresponding dilutions are listed in Table S2. Images were randomly captured and analysed using ImageJ software (NIH). The numbers of F4/80^+^ or CD68^+^ cells were counted and normalized by total cells.

### 2.17 RBC‐EV electroporation

The electroporation of RBC‐EVs was performed using a BTX electroporator (Harvard Biosciences, Cambridge, MA, USA). Briefly, 100 μg purified RBC‐EVs (100 μg ≈ 2.3 × 10^10^ particles) and 2.5 nmol FAM‐labelled scrambled NC‐ASOs or 5 nmol miR‐155‐ASOs with a 2′‐O‐methyl modification on every nucleotide and 3′ phosphorothioate internucleotide linkages at the first three 5′ and 3′ (Genepharma, Shanghai, China) were mixed in 100 μl of electroporation buffer (Harvard Biosciences). An exponential program was performed at a fixed capacitance of 100 μF to obtain the optimum efficiency in 0.2 cm cuvettes. The sequences for miR‐155‐ASOs and NC‐ASOs are listed in Table S3. For loading of the EVs with Dox or SRF, 100 μg purified EVs and 50 μg Dox or 100 μg SRF were gently mixed. After electroporation at 350 V and 150 μF in 0.4 cm electroporation cuvettes (Usman et al., 2018), the mixture was incubated at 37°C for 30 min to ensure that the plasma membrane of the EVs had fully recovered (Tian et al., 2014). For removal of the unincorporated free ASOs, Dox or SRF, the EVs were then washed with cold PBS twice prior to ultracentrifugation at 120,000 × *g* for 90 min. For determination of the loading efficiency, electroporated mixtures washed in PBS were placed in a 96‐well black plate with a clear bottom (PerkinElmer), and the FAM fluorescence was detected using a SynergyMx M5 plate reader (PerkinElmer) with excitation/emission at 485 nm/525 nm. For evaluation of RNase resistance, unelectroporated or washed electroporated mixtures were incubated with 100 units of RNase I (NEB, Beijing, China) at 37°C for 4 h. In some experiments, RBC‐EVs were pretreated with 1% Triton on ice for 1 h. The EVs loaded with Dox or SRF were quantified by high‐performance liquid chromatography (HPLC). Dox‐ or SRF‐loaded RBC‐EVs were ultrasonicated and then centrifuged at 120,000 × *g* for 90 min. The supernatant was separated and used for the LC‐MS/MS assay. The concentration of Dox or SRF in the EVs was determined using a previously reported LC‐MS/MS method (Heinz et al., 2011; Wei, Xiao, Si, & Liu, 2008).

### 2.18 Histology

Mouse lung, liver and kidney tissues were fixed with 10% paraformaldehyde, and sectioned into tissue blocks with a thickness of 5 μm. After routine dehydration, paraffin embedding, and serial sectioning, the paraffin‐embedded sections were stained with haematoxylin and eosin (H&E). The severity of the lung injury was graded based on oedema, congestion, neutrophil infiltration, haemorrhage, hyaline membranes and alveolar septal thickening (Zhou et al., 2019). Liver injury was assessed based on haemorrhage, hepatocyte necrosis, inflammatory cell infiltration, cytoplasm vacuolization and nuclear condensation (Yan et al., 2017). These scoring procedures were scored by two blinded observers. The average score in each group was calculated by the sum of the scores from each tissue divided by the number of tissues examined. These characteristics were subjectively scored on a scale from 0 to 4: 1, light; 2, moderate; 3, strong; 4, intense. The overall injury scores in each group were calculated by the sum of the scores from all the indicators.

### 2.19 Serum analysis

Blood was centrifuged at 1500 × *g* for 10 min to collect clear serum and analysed within 2 h. The serum activities of alanine transaminase (ALT), aspartate aminotransferase (AST), kinase isoenzyme‐MB (CK‐MB) and lactate dehydrogenase (LDH) were measured using the ALT, AST, CK‐MB and LDH Reagent Kits (Jiancheng, Nanjing, Jiangsu, China), respectively. The serum levels of tumour necrosis factor‐α (TNF‐α), interleukin‐1β (IL‐1β) and interleukin‐6 (IL‐6) were measured by ELISAs (Thermo Fisher Scientific) according to the manufacturer's instructions.

### 2.20 Cell viability assay

B16F10‐Luci or HCC‐LM3‐Luci cells (2 × 10^5^ cells/ml) were seeded in 96‐well plates and allowed to attach for 12 h. Then, 0.3 μg/ml Dox, 5 μg/ml Dox, 10 μg/ml RBC‐EVs loaded with Dox, 1.5 μg/ml SRF or 10 μg/ml RBC‐EVs loaded with SRF was added. The viability was assayed at 24 h, 48 h or 72 h by using a Cell Counting Kit‐8 assay (TransGen, Beijing, China).

### 2.21 Endothelial tube formation assay

After treatment with 1.5 μg/ml SRF or 10 μg/ml RBC‐EVs loaded with SRF for 24 h, primary HUVECs were seeded (1 × 10^4^ cells/well) in a growth factor‐reduced Matrigel (BD Bioscience, San Jose, CA USA)‐coated ibidi plate and incubated for 4 h at 37℃. Then, the cells were dyed with 6.25 μg/ml calcein AM (PromoKine, Heidelberg, Baden‐Württemberg, Germany) for 15 min. Tube formation was examined under an inverted fluorescence microscope, photographed and analysed by angiogenesis analyser plug‐ins of ImageJ (NIH).

### 2.22 Statistical analysis

All statistical analyses were performed using GraphPad Prism 8.0 (GraphPad Software Inc., San Diego, CA, USA). All data were expressed as the mean ± standard deviation. For normally distributed data, significance of mean differences was determined using unpaired Student's *t*‐test between two groups or ANOVA followed by Newman‐Keuls multiple comparison test among multiple groups. For data that were not normally distributed, nonparametric Kruskal‐Wallis H test were used for analysis. A difference was considered statistically significant if the *P* value was < 0.05.

## 3 RESULTS

### 3.1 Red blood cell extracellular vesicles naturally accumulate in the liver after intravenous injection

**FIGURE 1:**
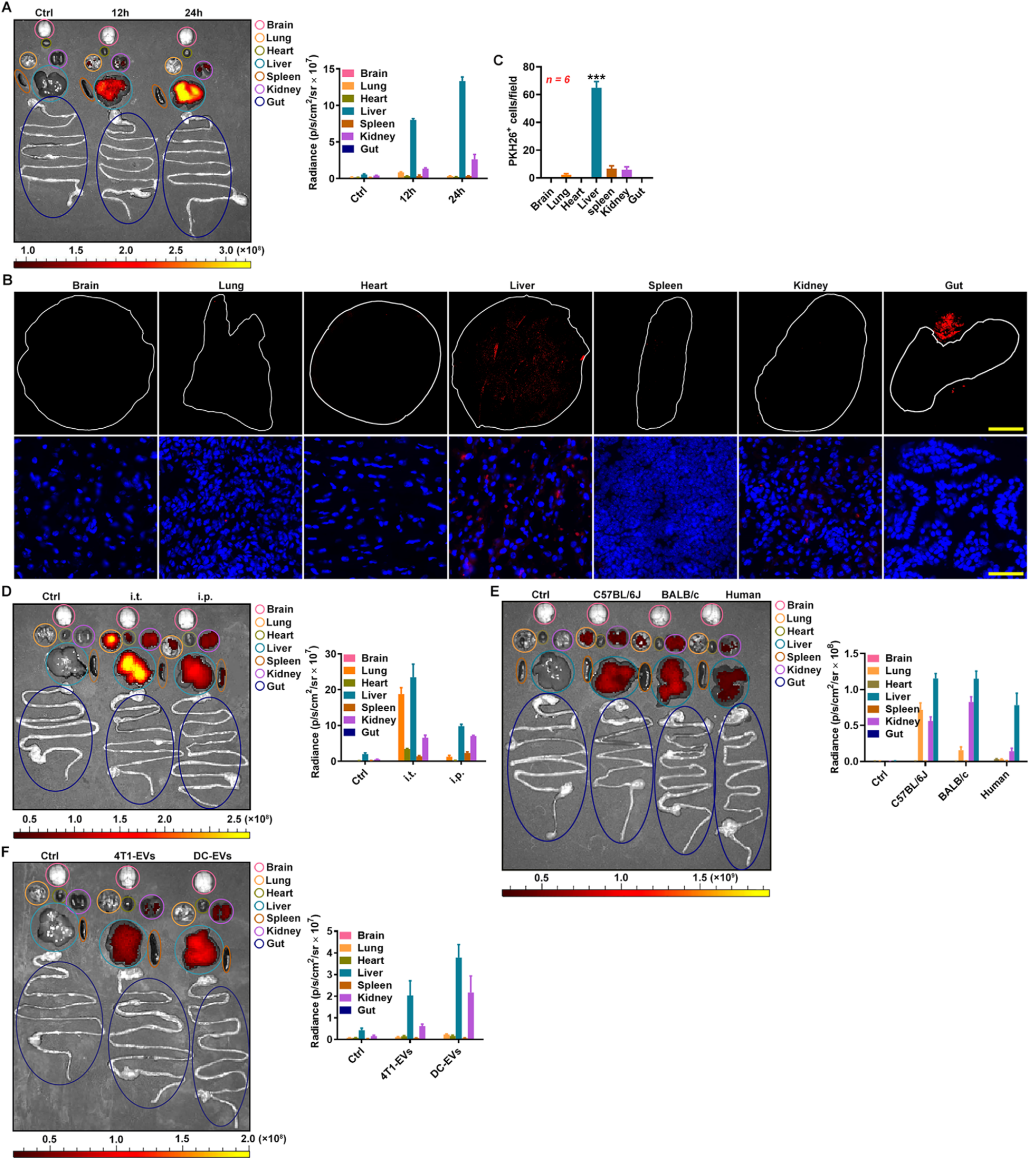
RBC‐EVs accumulate in the liver after intravenous injection. (A) Representative ex vivo imaging and quantification of organs from the C57BL/6J mice intravenously injected with 100 μg VivoTrack 680‐labelled C57BL/6J RBC‐EVs (≈ 2.3 × 10^10^ particles) for 12 or 24 h. (B, C) Full scanning imaging (top) or fluorescence microscopy detection (bottom) (B) and statistical analysis of PKH26^+^ cell numbers (C) of the indicated organ sections from the C57BL/6J mice injected with 100 μg PKH26‐labelled C57BL/6J RBC‐EVs. Scale bars, 2000 μm (top) and 50 μm (bottom) (B). (D) Representative ex vivo imaging and quantification of organs from the C57BL/6J mice that received intrathoracic or intraperitoneal injection with 100 μg VivoTrack 680‐labelled C57BL/6J RBC‐EVs for 24 h. (E) Representative ex vivo imaging and quantification of organs from the C57BL/6J mice intravenously injected with 100 μg VivoTrack 680‐labelled C57BL/6J, BALB/c or human RBC‐EVs for 24 h. (F) Representative ex vivo imaging and quantification of organs from the C57BL/6J mice intravenously injected with 100 μg VivoTrack 680‐labelled 4T1‐EVs or DC‐EVs for 24 h. ***P < 0.001, versus other groups (one‐way ANOVA followed by Newman‐Keuls multiple comparison test). Representative results from three independent experiments are shown

Given the excellent clinical application prospects of RBC‐EVs, we further evaluated their distribution in vivo. First, we separated and identified them. The RBC‐EVs we separated were negative for the microvesicle marker ARF6 (Jeppesen et al., 2019), positive for the RBC marker haemoglobin A1, and enriched for the ESCRT proteins Alix and Tsg101, and cytoplasmic protein HSP70 (Figure S1*A*). Visualized under transmission electron microscopy, the RBC‐EVs were vesicles approximately 100–150 nm in diameter with typical bilayer membrane structure (Figure S1*B*). Size distribution analysis revealed that the mean size of the RBC‐EVs was 154 ± 51 nm (Figure S1*C*). Then, the VivoTrack 680‐labelled RBC‐EVs were injected through the tail vein of the mice to assess their distribution in vivo. The RBC‐EVs accumulated mostly in the liver with some distribution in the kidneys when observed using an in vivo imaging system (IVIS) system (Figure 1A); these results were not obtained when VivoTrack 680 alone was injected (Figure S1*D*). These findings were confirmed by the fluorescence detection of various tissue sections after the mice were intravenously injected with the PKH26‐labelled RBC‐EVs (Figure 1B, C). Amphiphilic lipid‐anchored fluorophore compounds can form EV‐like particles (Simonsen, 2019), which most likely disturb the detection of the true distribution of the RBC‐EVs in vivo. To exclude this, we also detected the RBC‐EV distribution in vivo after the mice were intravenously injected with the RBC‐EVs labelled by CFSE, which labels proteins. In this way, we observed the accumulation of the RBC‐EVs in the liver (Figure S1*E, F*). In addition, the RBC‐EVs concentrated by sucrose gradient (SG‐RBC‐EVs) accumulated in the liver (Figure S1*D*), excluding this effect due to non‐EV contamination. The liver is one of the organs with the richest blood flow, and the accumulation of the RBC‐EVs in the liver may be caused by the anatomical characteristics of the liver following intravenous injection of the RBC‐EVs. Therefore, we also administered the RBC‐EVs by intrathoracic or intraperitoneal injection. Intrathoracic injection led to strong lung and moderate heart accumulation of the RBC‐EVs, and intraperitoneal injection caused the moderate spleen and lung accumulation of the RBC‐EVs (Figure 1D). However, neither intrathoracic injection nor intraperitoneal injection changed the trend towards the liver accumulation of RBC‐EVs (Figure 1D). Allogeneic blood is used for transfusions in the clinic. Subsequently, we evaluated the distribution of the RBC‐EVs from allogeneic mice in vivo and found that the RBC‐EVs from BALB/c mice showed a similar distribution in C57BL/6 mice (Figure 1E). Furthermore, the RBC‐EVs from humans displayed the same distribution in C57BL/6 mice (Figure 1E). In addition, we found that EVs from murine 4T1 breast tumour cells (4T1‐EVs) or bone marrow‐derived dendritic cells (DC‐EVs) also accumulated largely in the liver after intravenous injection, suggesting that liver accumulation is probably a common feature of EVs (Figure 1F). These results demonstrate that EVs will naturally accumulate in the liver after intravenous injection. Based on the yield and excellent biosafety (Usman et al., 2018), the RBC‐EVs from humans were used for subsequent experiments.

### 3.2 Liver accumulation of the RBC‐EVs is macrophage dependent

**FIGURE 2:**
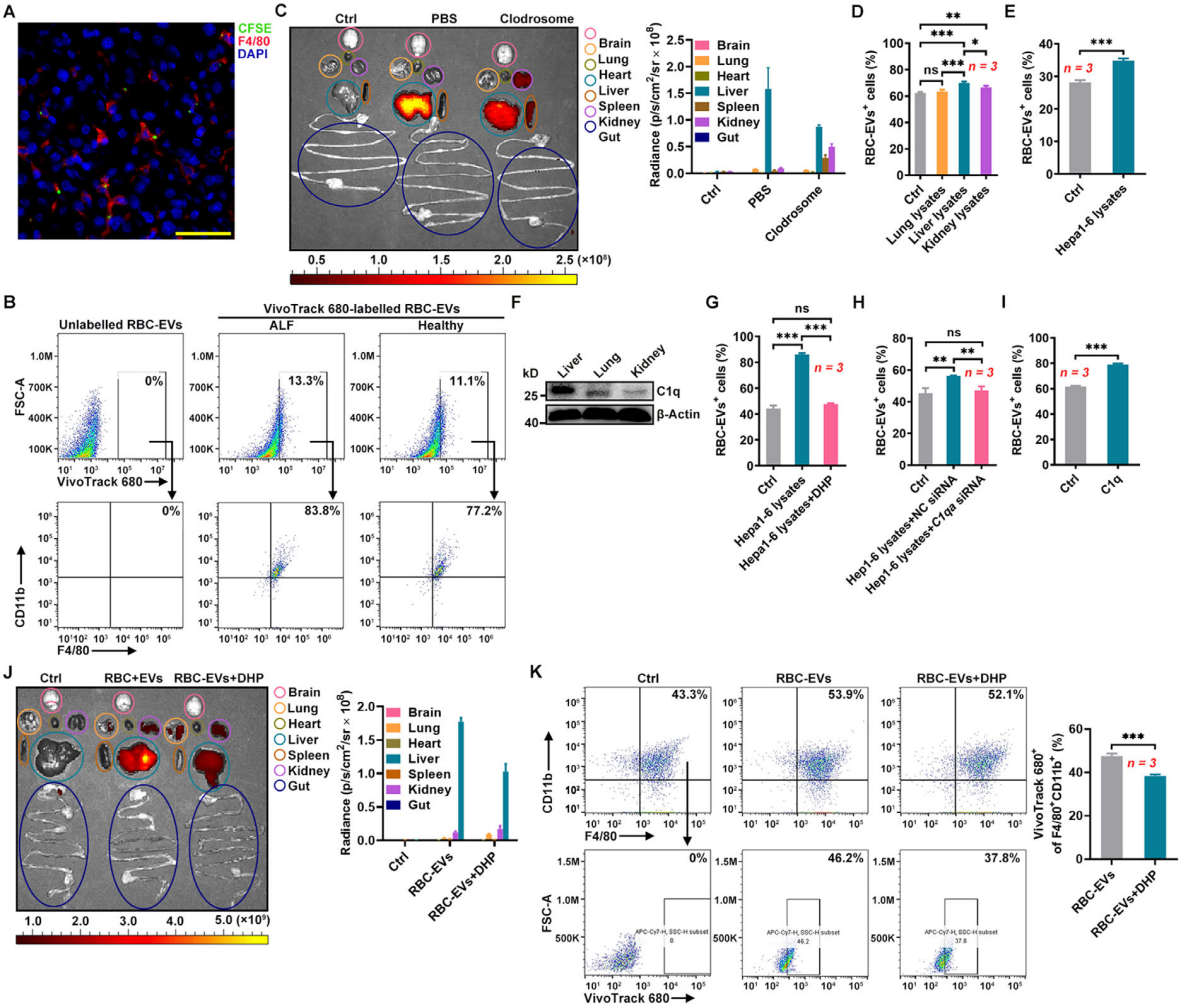
The liver accumulation of the RBC‐EVs is macrophage dependent. (A, B) D‐GalN and LPS (D‐GalN/LPS)‐induced mice with ALF were intravenously injected with 100 μg CFSE‐labelled RBC‐EVs (≈ 2.3 × 10^10^ particles) for 24 h. The fusion of RBC‐EVs with F4/80^+^ macrophages in livers was detected by fluorescence microscopy. Scale bars, 50 μm (A), and the uptake of RBC‐EVs by F4/80^+^CD11b^+^ macrophages in the liver was detected by flow cytometry (B). (C) Healthy mice were intravenously injected with 50 μl Clodrosomes for 24 h. Then, the mice were intravenously injected with 100 μg VivoTrack 680‐labelled RBC‐EVs for 24 h. Representative ex vivo imaging and quantification of organs from these mice are shown. (D) PMs were treated with 100 μg/ml lysate from lung, liver or kidney tissues for 24 h. Then, the cells were cultured with 20 μg/ml PKH26‐labelled RBC‐EVs for 6 h. The uptake of RBC‐EVs was detected by flow cytometry. (E) PMs were treated with 100 μg/ml Hepa1‐6 cell lysate for 24 h. Then, the cells were cultured with 20 μg/ml PKH26‐labelled RBC‐EVs for 6 h, and the uptake of RBC‐EVs was detected by flow cytometry. (F) C1q in liver, lung and kidney lysates was detected by western blotting. (G) PMs were stimulated with 100 μg/ml lysate from Hepa1‐6 cells that received 24 h treatment with or without 2.5 mM DHP. Twenty‐four hours later, the cells were cultured with 20 μg/ml PKH26‐labelled RBC‐EVs for 6 h and the uptake of the RBC‐EVs was detected by flow cytometry. (H, I) PMs were stimulated with 100 μg/ml lysate from Hepa1‐6 cells transfected with NC or *C1qa* siRNA (H), or 10 μg/ml C1q proteins for 24 h (I). Twenty‐four hours later, the cells were cultured with 20 μg/ml PKH26‐labelled RBC‐EVs for 6 h, and the uptake of RBC‐EVs was detected by flow cytometry. (J, K) Healthy mice were intraperitoneally injected with 250 mg/kg DHP for 24 h. Then, the mice were intravenously injected with 100 μg VivoTrack 680‐labelled RBC‐EVs for 24 h. Representative ex vivo imaging and quantification of organs from these mice are shown (J). The percentage of F4/80^+^CD11b^+^ macrophages taking up RBC‐EVs in the pre‐isolated KCs was detected by flow cytometry (K). ns, not significant; *P < 0.05; **P < 0.01 and ***P < 0.001 (one‐way ANOVA followed by Newman‐Keuls multiple comparison test in D, G and H or unpaired Student's *t*‐test in E, I and K). Representative results from two or three independent experiments are shown (mean and S.D.)

Then, we wanted to determine which liver cells are responsible for the liver accumulation of the RBC‐EVs. Hepatic stellate cells (HSCs), which account for 5%‐8% of liver cells (Blaner et al., 2009), might mediate the liver accumulation of the RBC‐EVs. However, only activated HSCs express the αSMA marker (Huang, Deng, & Liang, 2017). To collectively analyse the liver cells that take up the RBC‐EVs, we induced mice with ALF, in which HSCs express αSMA (Jin et al., 2017). First, we confirmed that the RBC‐EVs still showed liver accumulating in the mice with ALF, although stronger fluorescent signals could be observed in the liver and kidneys of these mice (Figure S2*A*). We found that αSMA^+^ HSCs did not fuse with the RBC‐EVs, nor did CD31^+^ endothelial cells or Albumin^+^ hepatocytes by immunofluorescence (Figure S2*B*). However, over 80% of the liver cells that took up the RBC‐EVs were F4/80^+^ according to immunofluorescence or were F4/80^+^CD11b^+^ macrophages according to flow cytometry (Figure 2A, B). We also confirmed that liver macrophages were the major type of liver cells that took up the RBC‐EVs in healthy mice (Figure 2B). Upon the depletion of macrophages by Clodrosome (Figure S2*C*), the liver accumulation of the RBC‐EVs was largely decreased, and the spleen and kidney concentrations of the RBC‐EVs was increased (Figure 2C). These results indicate that macrophages are involved in the liver accumulation of the RBC‐EVs.

After binding to its ligand CD47, signal regulatory protein alpha (SIRPα) can release the ‘don't eat me’ signal that inhibits phagocytosis (Logtenberg, Scheeren, & Schumacher, 2020). We assumed that liver macrophages ‘eat’ more RBC‐EVs due to the low levels of SIRPα. We indeed found that the relative *Sirpa* mRNA level in liver tissues was very low (Figure S2*D*). In addition, there were fewer SIRPα^+^ macrophages in the liver than in the lungs and spleen (Figure S2*E*). However, we could not detect CD47 on the RBC‐EVs (Figure S2*F*). These results indicate that the ‘don't eat me’ signal is likely not involved in the regulation of uptake of RBC‐EVs by liver macrophages.

We also detected CD47 on the 4T1‐EVs and the DC‐EVs and found both EVs contained very low amount of CD47 (Figure S2*G*). Given that the 4T1‐EVs and DC‐EVs also accumulated in the liver, we hypothesized that the liver‐specific environment contributed to enhanced phagocytosis of macrophages. Importantly, liver lysates greatly induced RAW264.7 macrophages and peritoneal macrophages (PMs) to phagocytize the RBC‐EVs (Figure S2*H* and Figure 2D). However, kidney lysates or lung lysates only slightly promoted the phagocytosis of the RBC‐EVs by RAW264.7 macrophages and PMs (Figure S2*H* and Figure 2D). In addition, ALF further enhanced the ability of liver lysates to promote PM phagocytosis of the RBC‐EVs (Figure S2*I*). We found that lysates of Hepa1‐6 murine liver tumour cells could also increase the phagocytosis of the RBC‐EVs by RAW264.7 macrophages and PMs (Figure S2*J* and Figure 2E). Liver is the primary site of complement synthesis, and C1q has been reported to enhance phagocytosis of macrophages (Espericueta, Manughian‐Peter, Bally, Thielens, & Fraser, 2020; Ghebrehiwet, Hosszu, & Peerschke, 2017). We found that the C1q level was higher in the liver lysates than in the kidney lysates and the lung lysates (Figure 2F). Then, we determined the role of C1q in the increased phagocytosis of macrophages. 3,4‐Dehydro‐d,l‐proline is an inhibitor of C1q synthesis (Leu, Stewart, Herriott, Fast, & Rummage, 1993). We confirmed that 3,4‐dehydro‐l‐proline (DHP) could also inhibit C1q synthesis in Hepa1‐6 cells (Figure S2*K*). After the inhibition of C1q, we found that the Hepa1‐6 cell lysates could no longer promote the phagocytosis of the RBC‐EVs by PMs any more (Figure 2G). We also did not observe increased phagocytosis of RBC‐EVs by PMs after treatment with the lysates from Hepa1‐6 cells with C1qA knockdown (Figure S2*L* and Figure 2H). More directly, C1q proteins substantially increased the phagocytosis of the RBC‐EVs by PMs (Figure 2I). In addition, intraperitoneal injection of DHP substantially inhibited the C1q protein levels in the liver (Figure S2*M*). Accordingly, mice with DHP administration showed decreased liver, and increased lung and kidney accumulation of the RBC‐EVs (Figure 2J). Furthermore, the percentage of liver macrophages taking up the RBC‐EVs was significantly reduced after DHP administration (Figure 2K). Altogether, our data suggest that C1q induces liver macrophages to phagocytize RBC‐EVs.

Furthermore, we observed more macrophages in the liver than in the lungs or kidneys, and the macrophages were further enriched in the liver of the mice with ALF, which probably contributes to the liver accumulation of the RBC‐EVs (Figure S2*N*).

### 3.3 RBC‐EVs show accumulation in human liver

**FIGURE 3:**
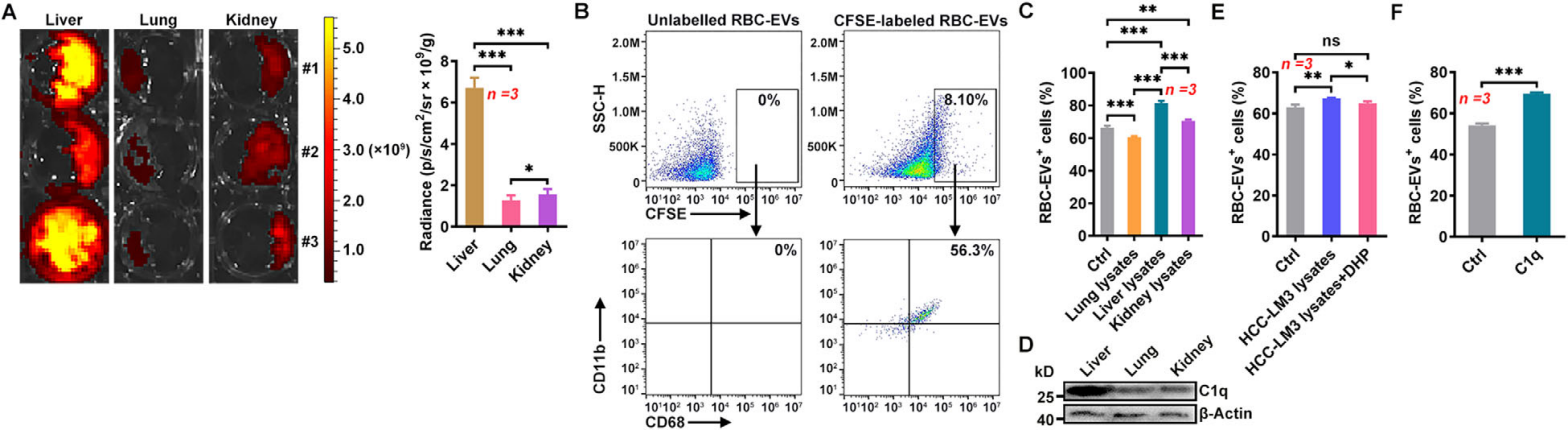
The RBC‐EVs show human liver accumulation. (A) Representative imaging and quantification of liver, lung and kidney tissue masses cultured with 20 μg/ml VivoTrack 680‐labelled RBC‐EVs for 24 h in vitro. (B) The liver tissue mass was cultured with 20 μg/ml CFSE‐labelled RBC‐EVs for 24 h in vitro, and then the uptake of RBC‐EVs by CD68^+^CD11b^+^ macrophages was detected by flow cytometry. (C) THP‐1 cells were stimulated with 50 ng/ml phorbol myristate for 48 h and then treated with 100 μg/ml lysate from lung, liver or kidney tissues. Twenty‐four hours later, the cells were cultured with 20 μg/ml PKH26‐labelled RBC‐EVs for 6 h, and the uptake of the RBC‐EVs was detected by flow cytometry. (D) C1q in liver, lung and kidney tissues was detected by western blotting. (E, F) THP‐1 cells were stimulated with 100 μg/ml lysate from HCC‐LM3 cells that received 24 h of treatment with or without 2.5 mM DHP (E), or 10 μg/ml C1q proteins (F). Twenty‐four hours later, the cells were cultured with 20 μg/ml PKH26‐labelled RBC‐EVs for 6 h, and the uptake of the RBC‐EVs was detected by flow cytometry. ns, not significant; *P < 0.05; **P < 0.01 and ***P < 0.001 (one‐way ANOVA followed by Newman‐Keuls multiple comparison test in A, C and E or unpaired Student's *t*‐test in F). Representative results from two or three independent experiments are shown (mean and S.D.)

To confirm that the RBC‐EVs could also accumulate in the human liver, we cultured small pieces of liver, lung and kidney tissues in medium containing VivoTrack 680‐labelled RBC‐EVs. We found that the RBC‐EVs accumulation was greater in the liver tissues than in the lung and kidney tissues (Figure 3A) and that over 56% of the liver cells that took up the RBC‐EVs were CD68^+^CD11b^+^ macrophages (Figure 3B). Then, we induced the conversion of human THP‐1 acute monocytic leukaemia cells to macrophages and found that the induced THP‐1 cells had a stronger ability to phagocytose the RBC‐EVs when treated with liver tissue than when treated with lung and kidney tissue lysates (Figure 3C). More C1q could also be detected in liver lysates than in kidney lysates and lung lysates (Figure 3D). Lysates of the human HepG2 and HCC‐LM3 liver cancer cells expressing C1q both increased the RBC‐EV phagocytosis of induced THP‐1 cells, which could be abolished by pretreatment of HepG2 and HCC‐LM3 cells with DHP (Figure S3*A, B* and Figure 3E). Again, C1q proteins substantially increased the phagocytosis of the RBC‐EVs by THP‐1 cells (Figure 3F). In addition, increases in CD68^+^ macrophages could be detected in liver tissues (Figure S3*C*). These results suggest that the human liver can recruit RBC‐EVs by increasing macrophage phagocytosis, which is also dependent on C1q.

### 3.4 RBC‐EVs loaded with antisense oligonucleotides of microRNA‐155 protect against ALF

**FIGURE 4:**
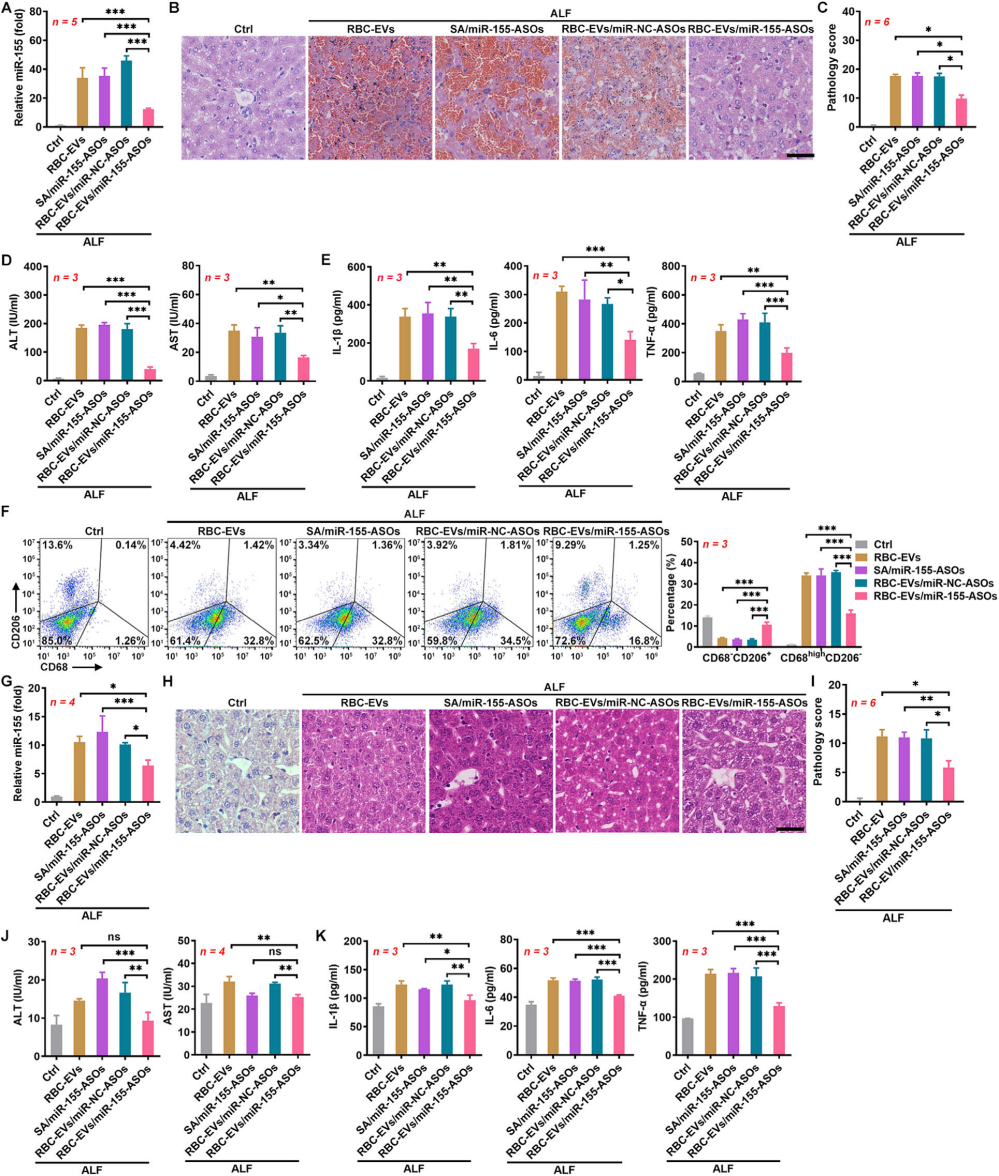
The RBC‐EVs loaded with miR‐155‐ASOs protect against ALF. (A‐F) Mice were intravenously injected with the SA/miR‐155‐ASOs (4 μg miR‐155‐ASOs), 100 μg RBC‐EVs/miR‐NC‐ASOs or RBC‐EVs/miR‐155‐ASOs (≈ 2.3 × 10^10^ particles) on days ‐3, ‐2 and ‐1, and then ALF was induced in these mice by D‐GalN/LPS on day 0. The mice were sacrificed, and the livers were isolated 24 h later. The miR‐155 levels in the livers were measured by real‐time PCR (A). Histopathological damage in the livers was detected by H&E staining. Scale bar, 40 μm (B), and the histopathological score was statistically analysed (C). The levels of ALT and AST in sera were measured (D). The levels of IL‐1β, IL‐6 and TNF‐α in sera were measured by ELISAs (E). CD68^–^CD206^+^ M2 and CD68^high^CD206^–^ M1 macrophages in livers were detected by flow cytometry (F). (G‐K) Six hours after ALF induction, mice were intravenously injected with the SA/miR‐155‐ASOs (4 μg miR‐155‐ASOs), 100 μg RBC‐EVs/miR‐NC‐ASOs or RBC‐EVs/miR‐155‐ASOs. Then, the mice were sacrificed 12 h later and the livers were isolated. The miR‐155 levels in the livers were measured by real‐time PCR (G). Histopathological damage in the livers was detected by H&E staining. Scale bar, 40 μm (H) and the histopathological score was statistically analysed (I). The levels of ALT and AST in sera were measured (J). The levels of IL‐1β, IL‐6 and TNF‐α in sera were measured by ELISAs (K). ns, not significant; *P < 0.05; **P < 0.01 and ***P < 0.001 (one‐way ANOVA followed by Newman‐Keuls multiple comparison test in A, D, E, F, G, J and K or Kruskal‐Wallis H test in C and I). Representative results from two or three independent experiments are shown (mean and S.D.)

Antisense oligonucleotides of microRNA‐155 (miR‐155‐ASOs) have been reported to alleviate liver injury with high efficiency (Yang et al., 2018). To examine whether the liver accumulation of RBC‐EVs could make them natural drug delivery vehicles for liver diseases, we loaded FAM‐labelled miR‐155‐ASOs into the RBC‐EVs by electroporation (RBC‐EVs/miR‐155‐ASOs) and observed their protective effect against ALF. First, we confirmed that electroporation and miR‐155‐ASO loading did not affect the integrity but slightly increased the size of the RBC‐EVs (Figure S4*A, B* and 1*C*). Then, we measured the FAM fluorescent intensity of the RBC‐EVs, and according to the standard curve for FAM fluorescent intensity and miR‐155‐ASO mass, we confirmed that 100 μg RBC‐EVs could encapsulate 4 μg miR‐155‐ASOs. We found that 10 μg RBC‐EVs/miR‐155‐ASOs containing 0.4 μg miR‐155‐ASOs but not the same amounts of miR‐155‐ASOs (SA/miR‐155‐ASOs) or RBC‐EVs loaded with miR‐NC‐ASOs (RBC‐EVs/miR‐NC‐ASOs) significantly reduced miR‐155 in LPS‐stimulated PMs (Figure S4*C*). We also concentrated the RBC‐EVs/miR‐155‐ASOs by a sucrose gradient (SG‐RBC‐EVs/miR‐155‐ASOs) and found that the SG‐RBC‐EVs/miR‐155‐ASOs showed an enhanced inhibitory effect on miR‐155 in the LPS‐stimulated PMs (Figure S4*D*). These results suggest that the RBC‐EVs/miR‐155‐ASOs but not the remaining miR‐155‐ASOs or non‐EV contamination mediate the inhibition of miR‐155. Moreover, the RBC‐EVs/miR‐155‐ASOs showed better resistance to RNase I than the SA/miR‐155‐ASOs (Figure S4*E*). Furthermore, after disruption by 1% Triton, the RBC‐EVs/miR‐155‐ASOs no longer exhibited resistance to RNase I (Figure S4*F*), suggesting that the RBC‐EVs promote the stability of the miR‐155‐ASOs. Importantly, the loading of the miR‐155‐ASOs did not affect the liver accumulation of the RBC‐EVs (Figure S4*G*). Preintravenous with the RBC‐EVs/miR‐155‐ASOs but not the SA/miR‐155‐ASOs strongly decreased the miR‐155 levels in the livers of the mice with ALF (Figure 4A). Consistently, the RBC‐EVs/miR‐155‐ASOs but not the SA/miR‐155‐ASOs produced striking protection against ALF upon the evaluation of liver histopathology, function and the levels of pro‐inflammatory cytokines (Figure 4B‐E). Silencing miR‐155 was shown to promote M2 but prevent M1 macrophage polarization (Zhang et al., 2016). Using CD68 and CD206 as reliable markers for M1/M2 macrophages (Nie et al., 2016), we then measured the relative abundance of M1 and M2 macrophages in the livers of the ALF group. We found that the RBC‐EVs/miR‐155‐ASOs but not the SA/miR‐155‐ASOs increased CD68^–^CD206^+^ M2 macrophages and decreased CD68^high^CD206^–^ M1 macrophages (Figure 4F). Consistently, the M2 macrophage marker genes *Arginase1* and *Il10* were significantly upregulated in the liver macrophages of the RBC‐EVs/miR‐155‐ASO‐treated mice along with substantial downregulation of the M1 macrophage marker genes *Il1b* and *iNos* (Figure S4*H*). Notably, similar protective effects against ALF were also obtained by the RBC‐EVs of C57BL/6J mice loaded with miR‐155‐ASOs (mRBC‐EVs/miR‐155‐ASOs) (Figure S4 *I‐M*). Altogether, these results suggest that the RBC‐EVs/miR‐155‐ASOs protect against ALF in a mouse model by regulating macrophage polarization.

Covalent attachment of a synthetic triantennary N‐acetylagalactosamine (GalNAc) ligand to chemically modified siRNA is an excellent approach for the delivery of therapeutic siRNA to hepatocytes in vivo (Brown et al., 2020). Therefore, we compared the protective effects of GalNAc conjugated miR‐155‐ASOs (GalNac‐miR‐155‐ASOs) and RBC‐EVs/miR‐155‐ASOs against ALF. The GalNac‐miR‐155‐ASOs showed a significant protective effect against ALF, but it was weaker than that of the RBC‐EVs/miR‐155‐ASOs (Figure S4*N‐R*). Consistent with their hepatocyte targeting, the GalNac‐miR‐155‐ASOs resulted in a greater decrease in miR‐155 in the F4/80^–^ cells than in the F4/80^+^ cells (Figure S4*S*). However, the RBC‐EVs/miR‐155‐ASOs showed a greater decrease in miR‐155 in the F4/80^+^ cells than in the F4/80^–^ cells (Figure S4*S*). These results suggest that the EVs/miR‐155‐ASOs have better protective effects against ALF than the GalNac‐miR‐155‐ASOs.

To further evaluate the prospects of the RBC‐EVs/miR‐155‐ASOs in clinical applications, we also investigated their therapeutic effects on ALF. The RBC‐EVs/miR‐155‐ASOs were administered 6 h after ALF induction. The RBC‐EVs/miR‐155‐ASOs but not the SA/miR‐155‐ASOs significantly decreased the miR‐155 levels in the livers of mice with ALF (Figure 4G). In addition, the RBC‐EVs/miR‐155‐ASOs but not the SA/miR‐155‐ASOs significantly ameliorated ALF as assessed by liver histopathology and function and the levels of pro‐inflammatory cytokines (Figure 4H‐K). These results conclude that the RBC‐EVs/miR‐155‐ASOs are effective in the treatment of ALF.

To test the specificity of the RBC‐EVs/miR‐155‐ASOs in the liver, we compared liver, lung and kidney injury in mice with LPS‐induced sepsis. The levels of miR‐155 in the livers, lungs and kidneys of septic mice all increased over time (Figure S5*A*). The RBC‐EVs/miR‐155‐ASOs significantly reduced the miR‐155 levels in the liver but not in the lungs and kidneys (Figure S5*B*). Moreover, the RBC‐EVs/miR‐155‐ASOs mitigated histopathological damage in the liver but not in the lungs (Figure S5*C, D*). We could not evaluate the protective effect of the RBC‐EVs/miR‐155‐ASOs on the histopathology of the kidneys because no obvious histopathological damage in the kidneys was observed in this model (Figure S5*C, D*).

### 3.5 The RBC‐EVs/miR‐155‐ASOs protect against ALF through a macrophage‐dependent mechanism

Given the RBC‐EVs/miR‐155‐ASOs were more effective in decreasing the miR‐155 levels in F4/80^+^ liver macrophages, the major cells that take up RBC‐EVs, than in F4/80^–^ liver cells in the mouse model of ALF (Figure S4*S*), we wanted to confirm the role of macrophages in the protective effects of the RBC‐EVs/miR‐155‐ASOs on ALF, we depleted the macrophages and found that the RBC‐EVs/miR‐155‐ASOs could still decrease the miR‐155 level in the liver and produce a protective effect against ALF (Figure S6*A‐E*). However, these effects were significantly weaker in the mice with macrophage depletion than in mice without macrophage depletion (Figure S6*A‐E*). Altogether, these results indicate that the RBC‐EVs/miR‐155‐ASOs specifically protect against liver injury through a mechanism is partially dependent on macrophages.

### 3.6 RBC‐EVs loaded with doxorubicin or sorafenib inhibit orthotopic liver cancer growth

**FIGURE 5:**
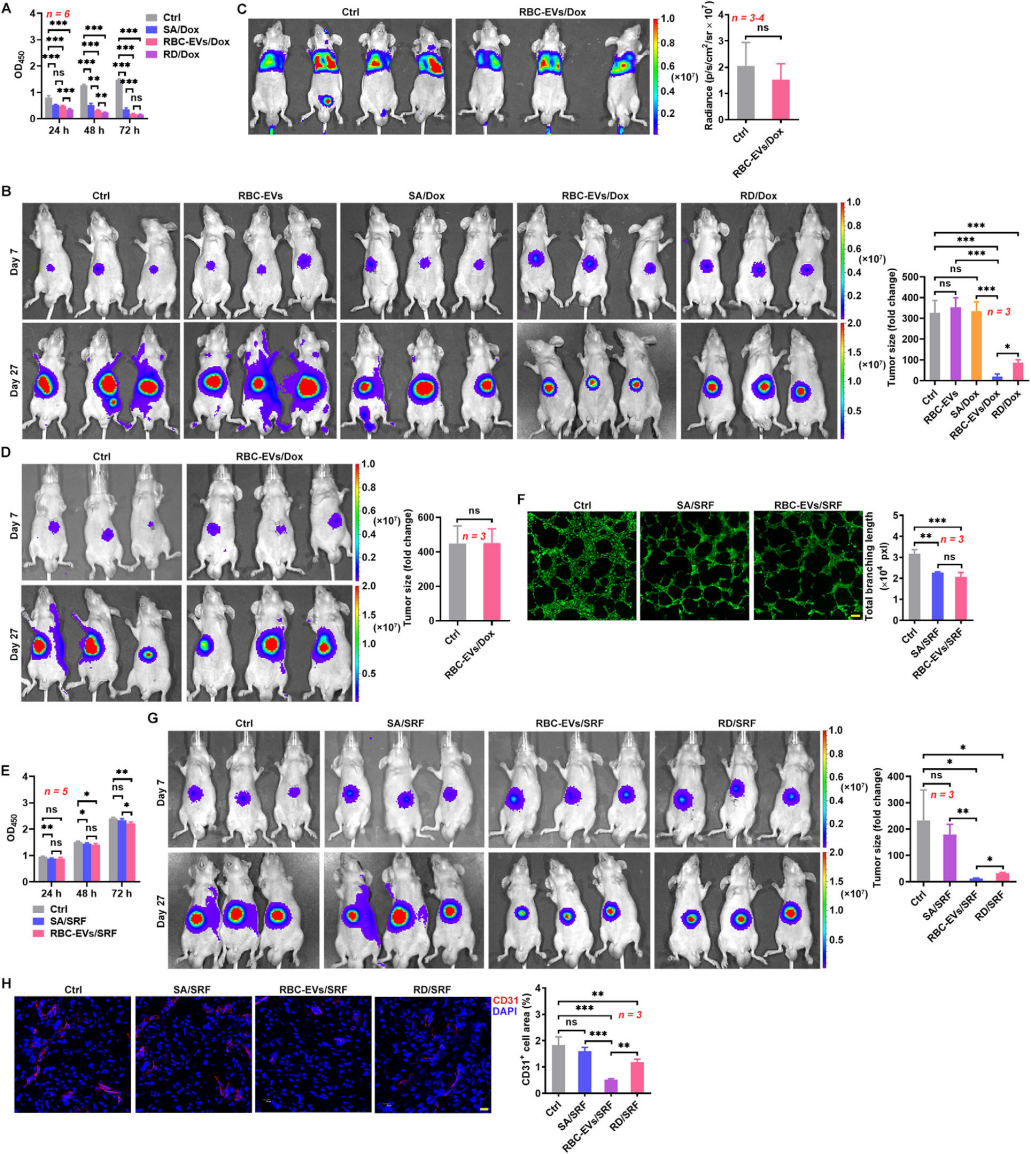
The RBC‐EVs loaded with Dox or SRF inhibit orthotopic liver cancer growth. (A) After treatment with SA/Dox (0.3 μg/ml Dox), RD/Dox (5 μg/ml) or 10 μg/ml RBC‐EVs/Dox for the indicated time, HCC‐LM3 cell viability was measured by a CCK‐8 assay. (B) HCC‐LM3‐Luci cells were orthotopically inoculated into mice on day 0. Then, the mice were intravenously injected with 100 μg RBC‐EVs (≈ 2.3 × 10^10^ particles), SA/Dox (3 μg Dox), RD/Dox (5 mg/kg Dox) or 100 μg RBC‐EVs/Dox every 3 days starting on day 7. The tumour size was monitored by IVIS on days 7 and 27 (left). Tumour progression was evaluated by calculating the tumour size on day 27 divided by that on day 7 (right). (C) Mice were intravenously injected with 1 × 10^6^ B16F10‐Luci cells on day 0. Then, the mice were intravenously injected with 100 μg RBC‐EVs/Dox every 3 days starting on day 7, and tumour progression was monitored by IVIS on day 21. (D) Tumour‐bearing mice were intravenously injected with 100 μg RBC‐EVs/Dox and 50 μl Clodrosomes (12 h before each RBC‐EVs/Dox injection) every 3 days starting on day 7. The tumour size was monitored (left), and the tumour progress was evaluated (right) as described in (B). (E) After treatment with SA/SRF (1.5 μg/ml SRF) or 10 μg/ml RBC‐EVs/SRF for the indicated time, HCC‐LM3 cell viability was measured by a CCK‐8 assay. (F) HUVECs were treated with SA/SRF or 10 μg/ml RBC‐EVs/SRF for 24 h followed by calcein AM staining. Then, tube formation was detected by fluorescence microscopy (left) and statistically analysed (right). Scale bars, 200 μm. (G, H) Tumour‐bearing mice were intravenously injected with SA/SRF (15 μg SRF), RD/SRF (30 mg/kg SRF) or 100 μg RBC‐EVs/SRF (≈ 2.3 × 10^10^ particles) every 3 days starting on day 7. The tumour size was monitored (left), and the tumour progress was evaluated (right) as described in (B) (G). Angiogenesis in tumour tissues was detected by CD31 staining (left), and the CD31^+^ cell area was statistically analysed (right). Scale bars, 20 μm (H). ns, not significant; *P < 0.05; **P < 0.01 and ***P < 0.001 (one‐way ANOVA followed by Newman‐Keuls multiple comparison test in A, B, E, F, G and H or unpaired Student's *t*‐test in C and D). Representative results from two or three independent experiments are shown (mean and S.D.)

To further confirm the effectiveness of RBC‐EVs as delivery vehicles for therapeutic drugs for liver diseases, we assessed the therapeutic effect of RBC‐EVs loaded with doxorubicin (RBC‐EVs/Dox) on orthotopic liver cancer. First, we evaluated the loading efficiency of Dox by the RBC‐EVs. According to the standard curve for Dox, the amount of Dox encapsulated into 100 μg RBC‐EVs was 3 μg (Figure S7*A, B*). We also confirmed that the stability of Dox in RBC‐EVs was excellent after 90 days (Figure S7*C*). Then, we examined the toxicity of the RBC‐EVs/Dox in HCC‐LM3 cells in vitro. We found that RBC‐EVs/Dox greatly inhibited HCC‐LM3 cell growth, and the effect was much stronger than that induced by the same amount of Dox (SA/Dox) and was even comparable to that of the routine dose of Dox (RD/Dox) (Figure 5A). We also concentrated the RBC‐EVs/Dox by a sucrose gradient (SG‐RBC‐EVs/Dox) and found the SG‐RBC‐EVs/Dox showed an enhanced inhibitory effect on HCC‐LM3 cell growth in vitro (Figure S7*D*). These results suggest that the RBC‐EVs/Dox but not the remaining Dox or non‐EV contamination mediate the most of the inhibitory effect. Then, orthotropic HCC‐LM3‐Luci tumours were established by the intrahepatic transplantation of tumour tissue from subcutaneous tumours into nude mice on day 0. When orthotopic tumours were observed on day 7, the mice were intravenously injected with the RBC‐EVs/Dox. As expected, whereas SA/Dox had no therapeutic effect on tumours, the RBC‐EVs/Dox showed a marked therapeutic effect that was even stronger than that of RD/Dox (Figure 5B). Similar inhibitory effects on tumours could also be obtained by RBC‐EVs of C57BL/6J mice loaded with Dox (mRBC‐EVs/Dox) (Figure S7*E*). The RBC‐EVs/Dox were also highly cytotoxic towards murine B16F10‐Luci melanoma cells in vitro (Figure S7*F*). However, compared with their effect on liver cancer, the RBC‐EVs/Dox had no therapeutic effect on metastatic lung B16F10 tumours (Figure 5C), suggesting the specificity of the effect of the RBC‐EVs/Dox on liver cancer. When detecting the distribution of Dox in liver cells, we found that Dox was mainly located in F4/80^+^ macrophages 6 h after the transfer of the RBC‐EVs/Dox but had dispersed into F4/80^–^ cells 12 h after the transfer of the RBC‐EVs/Dox (Figure S7*G*). Consistent with the distribution trend of Dox, the majority of the apoptotic cells were F4/80^+^ macrophages 12 h after the transfer of the RBC‐EVs/Dox (Figure S7*H*). However, 24 h after the transfer of the RBC‐EVs/Dox, many apoptotic F4/80^–^ cells were observed (Figure S7*H*). In addition, F4/80^+^ macrophages that could barely be detected 24 h after the transfer of the RBC‐EVs/Dox gradually appeared in the liver 48 h after the transfer of RBC‐EVs/Dox, suggesting the migration of macrophages from outside the liver (Figure S7*H*). These results suggest that Dox diffused into F4/80^–^ cells and induced apoptosis after its release from apoptotic F4/80^+^ macrophages. More importantly, we found that the depletion of macrophages completely abrogated the therapeutic effect of the RBC‐EVs/Dox on orthotopic liver cancer (Figure 5D). Therefore, these data indicate that the unique therapeutic effect of the RBC‐EVs/Dox on liver cancer occurs in a macrophage‐dependent manner.

SRF, which possesses both antitumor and antiangiogenic effects, is a first‐line drug for liver cancer (Cheng, Wei‐Qi, & Jin, 2020). Therefore, we also examined the therapeutic effect of RBC‐EVs loaded with SRF (RBC‐EVs/SRF) on orthotopic liver cancer. First, according to the standard curve of the SRF standard, the amount of SRF encapsulated in 1 μg RBC‐EVs was 0.15 μg (Figure S7*I, J*). Both the SA/SRF and the RBC‐EVs/SRF slightly inhibited HCC‐LM3 cell growth in vitro (Figure 5E). In addition, the SA/SRF and the RBC‐EVs/SRF both substantially inhibited tube formation in human umbilical vein endothelial cells (HUVECs) with the same efficiency (Figure 5F). Similar to the RBC‐EVs/Dox, the RBC‐EVs/SRF exhibited a better therapeutic effect on HCC‐LM3 orthotopic liver cancer than the RD/SRF, and the SA/SRF showed no therapeutic effect (Figure 5G). Both the RD/SRF and the RBC‐EVs/SRF, but not the SA/SRF, notably inhibited angiogenesis in tumour tissues (Figure 5H). However, in contrast to the in vitro results, the RBC‐EVs/SRF had an even stronger ability to inhibit angiogenesis than the RD/SRF (Figure 5H). These results indicate that the RBC‐EVs can effectively deliver therapeutic drugs for the treatment of liver cancer.

### 3.7 Toxicity evaluation of the drug‐loaded RBC‐EVs

**FIGURE 6:**
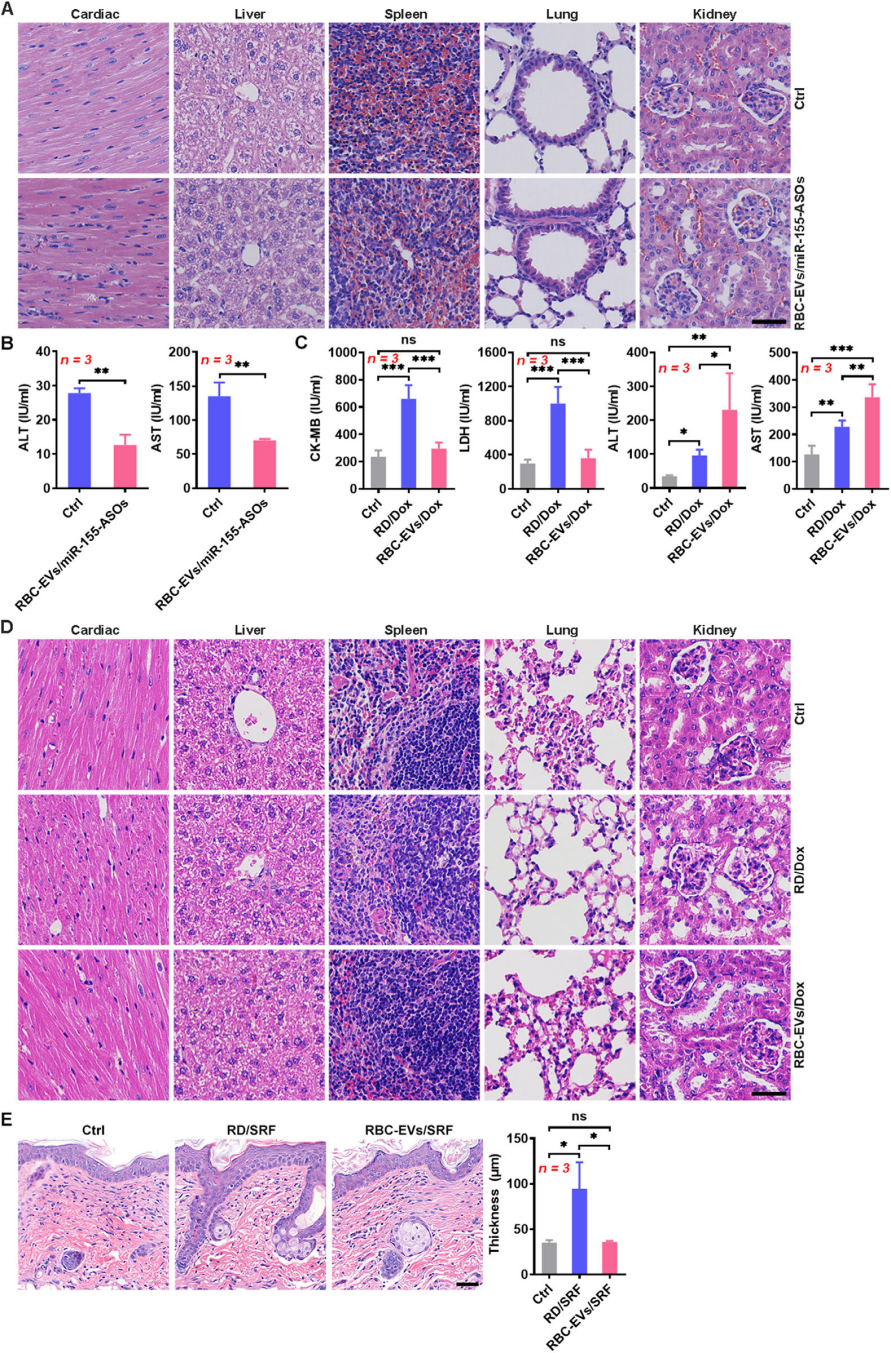
Toxicity evaluation of the drug‐loaded RBC‐EVs. (A, B) Mice were intravenously injected with 100 μg RBC‐EVs/miR‐155‐ASOs (≈ 2.3 × 10^10^ particles) on days ‐3, ‐2 and ‐1 and were sacrificed on day 1. Histopathological damage in the heart, liver, spleen, lungs and kidneys was detected by H&E staining. Scale bar, 40 μm (A). The levels of ALT and AST in sera were measured (B). (C, D) Mice were intravenously injected with RD/Dox (5 mg/kg Dox) or 100 μg RBC‐EVs/Dox (≈ 2.3 ×10^10^ particles) every 3 days starting on day 0. The levels of CK‐MB, LDH, ALT and AST in sera were measured (C), and histopathological damage in the heart, liver, spleen, lungs and kidneys was detected by H&E staining (D) on day 18. Scale bar, 40 μm. (E) Mice were intravenously injected with RD/SRF (30 mg/kg SRF) or 100 μg RBC‐EVs/SRF (≈ 2.3 ×10^10^ particles) every 3 days starting on day 0. Histopathological damage in the back skin was detected by H&E staining (left), and the thickness of the stratum spinosum was statistically analysed (right). Scale bar, 40 μm. ns, not significant; *P < 0.05; **P < 0.01 and ***P < 0.001 (unpaired Student's *t*‐test in *B* or one‐way ANOVA followed by Newman‐Keuls multiple comparison test in C and E). Representative results from three independent experiments are shown (mean and S.D.)

Biological safety is essential for the clinical application of drugs. The intravenous injection of the RBC‐EVs/miR‐155‐ASOs did not cause obvious histopathological damage to the main organs, including the heart, liver, spleen, lungs and kidneys (Figure 6A). Interestingly, even when accumulated in the liver, RBC‐EVs/miR‐155‐ASOs did not increase the levels of ALT and aspartate aminotransferase (AST) in sera; they decreased them (Figure 6B). The main side effect of Dox is cardiotoxicity (Singal & Iliskovic, 1998). The levels of kinase isoenzyme‐MB (CK‐MB) and LDH, which are markers of cardiac damage, in the serum of the mice that received intravenous injections of the RBC‐EVs/Dox did not significantly increase (Figure 6C). However, the RD/Dox led to significant cardiac damage (Figure 6C). Compared with the RD/Dox, the RBC‐EVs/Dox caused more severe impairment of liver function (Figure 6C), suggesting the improved accumulation of Dox in the liver by the RBC‐EVs/Dox. The RD/Dox also caused substantial histopathological damage in the heart but not in the spleen, lungs or kidneys, and the RBC‐EVs/Dox induced no obvious histopathological damage in the heart, spleen, lungs or kidneys (Figure 6D). Although the RD/Dox and the RBC‐EVs/Dox both impaired liver function, there was no obvious histopathological damage in the livers in either group of mice (Figure 6D). Furthermore, weight loss and weakness were only observed in mice that received the RD/Dox injection (Figure S8*A, B*). Neither the RBC‐EVs/SRF nor the RD/SRF affected angiogenesis in the heart, liver, spleen, lungs and kidneys (Figure S8*C, D*). SRF has been reported to cause severe skin toxicity (Ulrich, Hartmann, Dörr, & Ugurel, 2008). We found that skin from the RD/SRF‐treated mice showed increased thickness and downward extension of the stratum spinosum (Figure 6E). However, the RBC‐EVs/SRF treatment caused no histopathological damage to the skin (Figure 6E). These results demonstrate that when used for liver disease therapy, drug‐loaded RBC‐EVs do not cause systemic adverse effects.

## 4 DISCUSSION

Although EVs have been demonstrated to be potential delivery vehicles for therapeutic drugs, the organ‐ or cell‐specific targeting of EVs is a substantial obstacle that must be overcome. Many efforts have been made to improve the organ or cell‐specific targeting of EVs (Alvarez‐Erviti et al., 2011; Longatti et al., 2018; Tian et al., 2014). However, the artificial modification of EVs complicates their production process and restrains their large‐scale application. In addition, the modifications themselves likely cause unexpected side effects. For example, integrin αv‐specific iRGD peptide was used to target EVs to integrin αv‐positive breast cancer cells (Tian et al., 2014). However, integrin signalling can activate pro‐inflammatory *S100* gene expression, which is correlated with tumour metastasis (Hoshino et al., 2015). Rabies viral glycoprotein‐derived peptide, which can bind to its specific receptor (acetylcholine receptor), was used to target EVs to the brain (Alvarez‐Erviti et al., 2011). Whether the binding of rabies viral glycoprotein peptide will activate the acetylcholine receptor and the effects mediated by the activated receptor are unclear. RBC‐EVs show a natural liver accumulation that confers a unique biological safety profile. In addition, as drug delivery vehicles, EVs from red blood cells (RBC‐EVs) are safe. Because RBCs lack both nuclear and mitochondrial DNA, as do their parental cells, RBCs have been used safely and routinely for blood transfusions for decades (Usman et al., 2018). Therefore, RBC‐EVs are optimal drug deliverers for liver diseases.

The EV integrins α_6_β_4_ and α_6_β_1_ are associated with the lung tropism of EVs, and the EV integrin α_v_β_5_ is related to the liver tropism of EVs (Hoshino et al., 2015). Our previous publication has also demonstrated that EpCAM determines the localization of EVs from intestinal epithelial cells (Jiang et al., 2016). Therefore, EV characteristics play important roles in organ tropism. In this study, we found that liver accumulation is not a specific feature of RBC‐EVs, because 4T1‐EVs and dendritic cell‐EVs (DC‐EVs) also accumulated in the liver after intravenous administration. In previous study, macrophages were crucial for EV elimination from the systemic circulation including liver and spleen macrophages (Imai et al., 2015). Consistently, our results demonstrated that liver accumulation of RBC‐EVs mainly depended on macrophages. However, we further explored the mechanisms by which liver macrophages take up EVs and concluded that this phenomenon was probably due to three factors: first, signal regulatory protein alpha (SIRPα) expression on liver macrophages was lower than that on lung and spleen macrophages, probably resulting in weaker activation of the ‘don't eat me’ signal in liver macrophages; second, we found that highly abundant C1q in the liver could enhance the phagocytosis of RBC‐EVs by macrophages; third, the numbers of macrophages in the liver were higher than those in the lungs and spleen. All these factors will contribute to the uptake of EVs by liver macrophages and subsequent liver accumulation. It is worth mentioning that opsonization mediated by murine antibodies against antigens of human RBC‐EVs may also play a role in the fast uptake of RBC‐EVs by liver macrophages. However, this effect probably is minimum. Because liver accumulation could also be observed after murine RBC‐EVs were injected. Furthermore, drug‐loaded murine RBC‐EVs showed similar therapeutic effects on ALF and orthotopic liver cancer to drug‐loaded human RBC‐EVs.

The RBC‐EVs loaded with antisense oligonucleotides of microRNA‐155 (RBC‐EVs/miR‐155‐ASOs) but not the same amount miR‐155‐ASOs (SA/miR‐155‐ASOs) showed a distinct protective effect against ALF. Similarly, the RBC‐EVs loaded with doxorubicin (RBC‐EVs/Dox) but not the same amount Dox (SA/Dox) substantially inhibited orthotopic liver cancer growth. Furthermore, the therapeutic effect of the RBC‐EVs/Dox was even greater than that of the routine dose Dox (RD/Dox). The liver accumulation of the drug‐loaded RBC‐EVs led to the accumulation of drugs at high concentrations in the liver, which likely contributed to the prominent therapeutic effects of the drug‐loaded RBC‐EVs. In addition, RBC‐EVs themselves are responsible for the therapeutic advantages of drug‐loaded RBC‐EVs. For example, RBC‐EVs could protect the miR‐155‐ASOs from hydrolysis by RNase I, likely resulting in the improved stability of the miR‐155‐ASOs in vivo. In addition, tumour cell‐derived microparticles could also impede Dox evacuation (Tang et al., 2012). EV‐encapsulated paclitaxel has also been reported to overcome multiple drug resistance in cancer cells (Kim et al., 2016). It can be concluded that chemotherapeutic drugs encapsulated into RBC‐EVs are also highly cytotoxic towards tumour cells. Therefore, in addition to being simple delivery vehicles of drugs, RBC‐EVs have unique features that can also dramatically enhance the therapeutic effects of loaded drugs.

Macrophages are the main cells that produce pro‐inflammatory cytokines during ALF contributing to liver injury. The RBC‐EVs/miR‐155‐ASOs were mainly taken up by liver macrophages, which was beneficial for their protective effect against ALF. Inhibition of miR‐155 has been demonstrated to prevent pro‐inflammatory M1 while promoting anti‐inflammatory M2 macrophage polarization (Zhang et al., 2016). M2 macrophages are reported to ameliorate ALF by inhibiting hepatocellular apoptosis and promoting liver regeneration (Ito et al., 2017). Our results showed that the RBC‐EVs/miR‐155‐ASOs increased M2 but decreased M1 macrophages in livers of mice with ALF, thus rescuing the balance of M1 and M2 macrophage polarization. This phenomenon promotes the attenuation of ALF. Unexpectedly, although macrophages are crucial for ALF, macrophage depletion did not alleviate ALF. Liver macrophages are highly heterogeneous groups. They can be derived from either resident hepatic macrophages, called Kupffer cells (KCs) or from distinct populations of infiltrating macrophages, e.g. circulating bone marrow‐derived macrophages, avascular peritoneal macrophages that reside in the subcapsular regions of the liver or splenic monocytes (Van Der Heide, Weiskirchen, & Bansal, 2019). In response to liver microenvironmental signals, macrophages can polarize into M1 and M2 macrophages (Sica, Invernizzi, & Mantovani, 2014). If the anti‐inflammatory effects of M2 macrophages are equivalent to the pro‐inflammatory effects of M1 macrophages, they will counteract each other and thus, indiscriminate depletion of macrophages probably will not affect ALF. However, the collective effects of macrophages on ALF still need further study. After macrophage depletion, attenuated ALF could not be obtained, suggesting that in addition to macrophages, hepatocytes play an important role in ALF. In the presence of macrophages, both M1 macrophages and hepatocytes contributed to ALF, which was restrained by M2 macrophages. After intravenous injection with the RBC‐EVs/miR‐155‐ASOs, uptake of the RBC‐EVs/miR‐155‐ASOs by macrophages caused a strong decrease in miR‐155 in the liver, leading to decreased M1 and increased M2 macrophages in liver. Therefore, the pro‐inflammatory forces were reduced, while the anti‐inflammatory forces were enhanced, and notable protective effects on ALF were observed. In the absence of macrophages, uptake of the RBC‐EVs/miR‐155‐ASOs by hepatocytes was weak, reflected by the decreased miR‐155 in liver cells. Thus, RBC‐EVs/miR‐155‐ASOs treatment resulted in moderate protective effects on ALF. Alternatively, these results support the notion that macrophages are the main cells responsible for liver accumulation of the RBC‐EVs/miR‐155‐ASOs.

In a mouse model of liver cancer, we found that macrophages were also the main cells that captured the RBC‐EVs/Dox. After fusion with macrophages, Dox in RBC‐EVs induced macrophage apoptosis that led to the diffusion of Dox into other types of cells. In addition, these macrophages may have produced EVs with cytotoxicity, resulting in a cascade effect for tumour killing. Tumour cells killed by methotrexate‐encapsulating microparticles have been demonstrated to form new cytotoxic methotrexate‐packaging microparticles, leading to a cascade effect for tumour killing (Tang et al., 2012). Thus, RBC‐EVs/Dox are highly cytotoxic even when loaded with very low levels of Dox. Our results demonstrated that although the dose of Dox in the RBC‐EVs/Dox was approximately one‐thirty‐third of the RD/Dox, the RBC‐EVs/Dox still showed a stronger inhibitory effect on orthotopic liver cancer than the RD/Dox. With such low‐dose Dox, systemic immunosuppression that is common in patients treated with chemotherapy is probably avoidable. Therefore, stronger systemic antitumor immunity may be induced in tumour hosts with RBC‐EVs/Dox treatment, which may benefit the inhibition of not only primary tumours but also metastatic tumours. In this study, a T‐cell‐deficient nude mouse xenograft model of human liver cancer was used. Therefore, we could not assess the antitumor immune responses after RBC‐EVs/Dox treatment. In tumour hosts with a complete immune system, RBC‐EVs/Dox probably have better antitumor effects due to avoidable immunosuppression after treatment.

Collectively, our data revealed that RBC‐EVs show natural liver accumulation. After loading with drugs such as miR‐155‐ASOs, Dox or SRF, RBC‐EVs can specifically deliver drugs into the liver and produce potent therapeutic effects against ALF or liver cancer. More importantly, the use of drug‐loaded RBC‐EVs is feasible for clinical applications because of their excellent potential biological safety, high‐level production and simple production process. Thus, drug‐loaded RBC‐EVs are promising candidates for the treatment of liver diseases.

## CONFLICTS OF INTEREST

The authors report no conflicts of interest.

## Supporting information

Supporting information.

## Data Availability

The data for the study are available from the corresponding author upon request.
