# Supplementary material for: Extracellular vesicles: Natural liver‐accumulating drug delivery vehicles for the treatment of liver diseases
Source: J Extracell Vesicles. 2020 Dec 9;10(2):e12030. doi: 10.1002/jev2.12030 (PMC7726052; doi:10.1002/jev2.12030)
Supplement: Supplementary file 1 — Supporting information. [file JEV2-10-e12030-s001.docx]

**
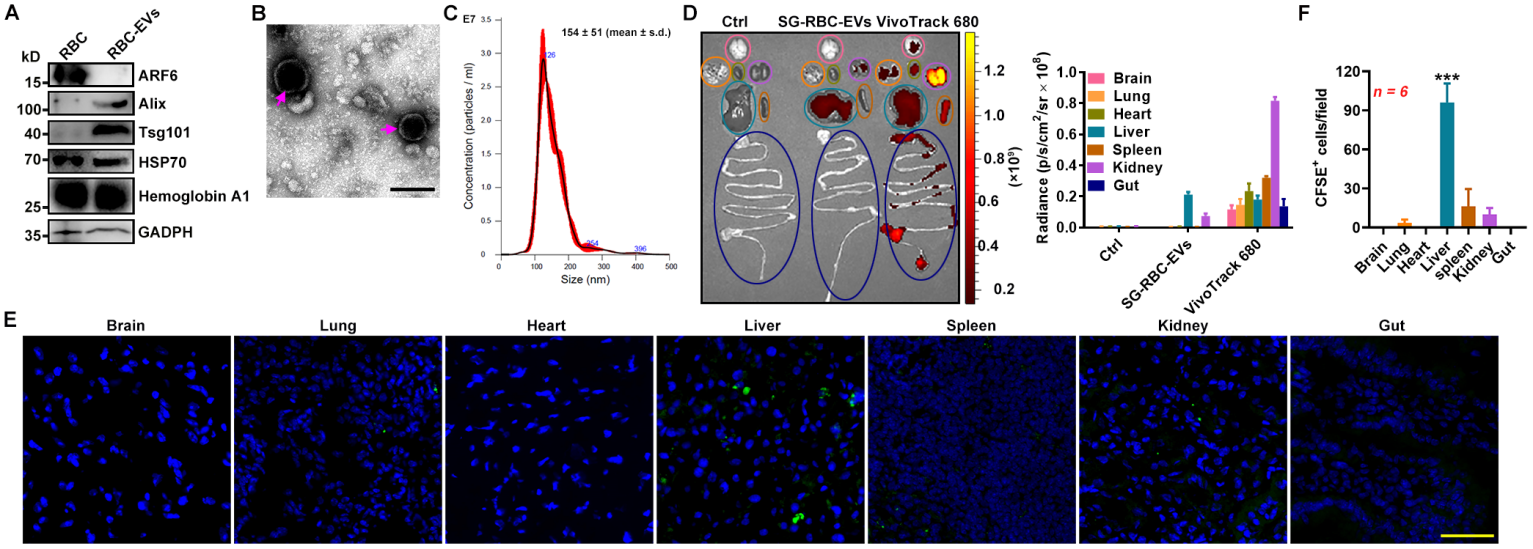
**

**Supplementary Figure 1: The RBC-EVs accumulate in the liver after intravenous injection**

(*A*) The levels of ARF6, Alix, HSP70, Tsg101 and Hemoglobin A1 relative to those of GAPDH (loading control) in RBC lysates and RBC-EVs were detected by western blotting. (*B*) The morphology of the RBC-EVs was detected by transmission electron microscopy. Arrows indicate EVs. Scale bar, 200 nm. (*C*) The size distribution of the RBC-EVs was analyzed by Nanoparticle Tracking Analysis. (*D*) Representative *ex vivo* imaging and quantification of organs from the C57BL/6J mice intravenously injected with 2 μg VivoTrack 680 or 100 μg VivoTrack 680-labeled SG-RBC-EVs (≈ 2.3 × 10^10^ particles) for 24 h. (*E*) Fluorescence microscopy detection of the indicated organ sections from the C57BL/6J mice injected with 100 μg CFSE-labeled RBC-EVs (≈ 2.3 × 10^10^ particles). Scale bar, 50 µm. (*F*) Statistical analysis of CFSE^+^ cell numbers in (*E*). ****P* < 0.001, versus other groups (one-way ANOVA followed by Newman-Keuls multiple comparison test). Representative results from three independent experiments are shown.

**
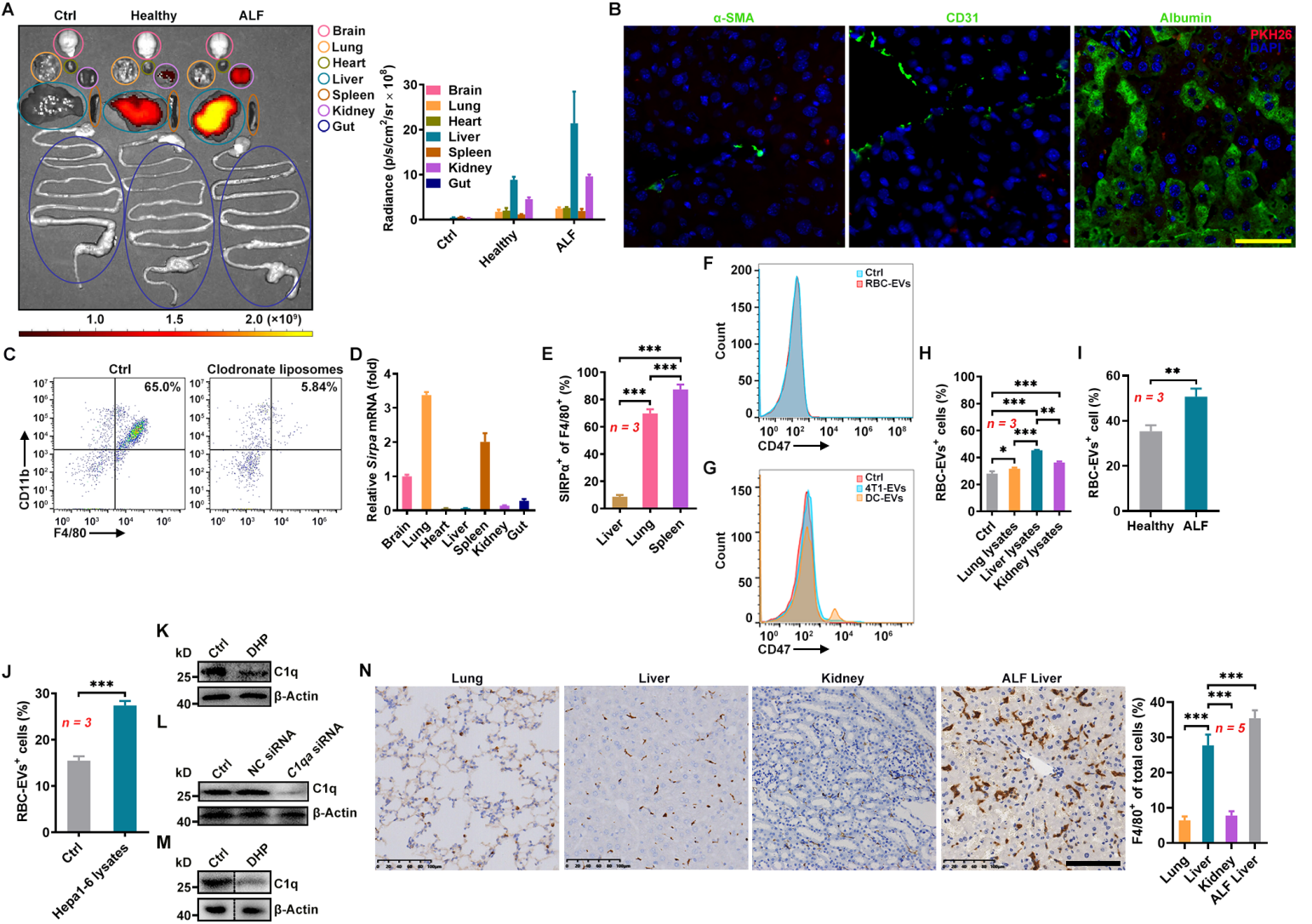
**

**Supplementary Figure 2: Liver tropism of the RBC-EVs is macrophage dependent**

1. Representative *ex vivo* imaging and quantification of organs from D-GalN/LPS-induced mice with ALF intravenously injected with 100 μg VivoTrack 680-labeled RBC-EVs (≈ 2.3 × 10^10^ particles) for 24 h. (*B*) The mice with ALF were intravenously injected with 100 μg PKH26-labeled RBC-EVs for 24 h. The fusion of the RBC-EVs with αSMA^+^ HSCs, CD31^+^ endothelial cells or Albumin^+^ hepatocytes in livers was detected by fluorescence microscopy. Scale bars, 50 µm. (*C*) Healthy mice were intravenously injected with 50 μl Clodrosomes for 24 h. Then, the F4/80^+^CD11b^+^ macrophages in pre-isolated KCs were detected by flow cytometry. (*D*) *Sirpa* mRNA levels in the indicated organs were detected by real-time PCR. (*E*) The percentage of SIRPa^+^ cells among the F4/80^+^ cells was analyzed by flow cytometry. (*F, G*) CD47 on the RBC-EVs (*F*), 4T1-EVs or DC-EVs (*G*) was detected by flow cytometry. (*H*) RAW264.7 macrophages were treated with 100 μg/ml lysate from lung, liver or kidney tissues for 24 h. Then, the cells were cultured with 20 μg/ml PKH26-labeled RBC-EVs for 6 h. The uptake of the RBC-EVs was detected by flow cytometry. (*I*) PMs were treated with 100 μg/ml liver lysate of healthy or mice with ALF for 24 h. Then, the cells were cultured with 20 μg/ml PKH26-labeled RBC-EVs for 6 h, and the uptake of the RBC-EVs was detected by flow cytometry. (*J*) RAW264.7 macrophages were treated with 100 μg/ml lysate from Hepa1-6 cells for 24 h. Then, the cells were cultured with 20 μg/ml PKH26-labeled RBC-EVs for 6 h, and the uptake of the RBC-EVs was detected by flow cytometry. (*K*) C1q in Hepa1-6 cells treated with or without 2.5 mM DHP for 24 h was detected by western blotting. (*L*) C1q in Hepa1-6 cells transfected with negative control (NC) or *C1qa* siRNA for 24 h was detected by western blotting. (*M*) Healthy mice were intraperitoneally injected with 250 mg/kg DHP for 24 h and then the C1q in the livers was detected by western blotting. Dotted lines indicate the bands are not adjacent (*N*) F4/80^+^ cells in the lungs, liver, kidneys and liver of mice with ALF were detected by immunohistochemistry. Scale bars, 100 µm. ns, not significant; **P* < 0.05; ***P* < 0.01 and ****P* < 0.001 (one-way ANOVA followed by Newman-Keuls multiple comparison test in *E*, *H* and *N* or unpaired Student’s *t*-test in *I* and *J*) . Representative results from three independent experiments are shown (mean and s.d.).

**
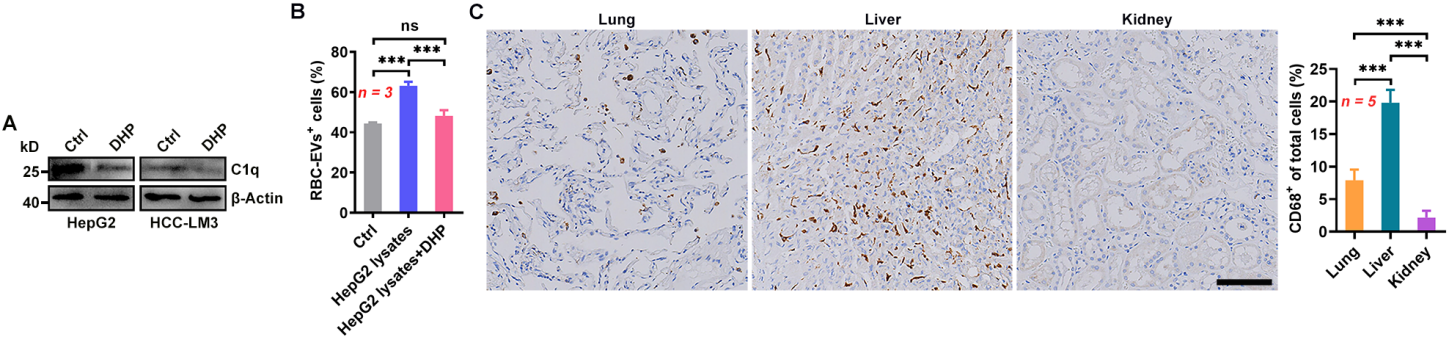
**

**Supplementary Figure 3: Liver is enriched in macrophages**

(*A*) C1q in HepG2 and HCC-LM3 cells treated with or without 2.5 mM DHP for 24 h was detected by western blotting. (*B*) THP-1 cells were stimulated with 100 μg/ml lysate from HepG2 cells that received 24 h of treatment with or without 2.5 mM DHP. Twenty-four hours later, the cells were cultured with 20 μg/ml PKH26-labeled RBC-EVs for 6 h, and the uptake of the RBC-EVs was detected by flow cytometry. (*C*) CD68^+^ macrophages in lung, liver and kidney tissues were detected by immunohistochemistry. Scale bars, 100 µm. ns, not significant; ****P* < 0.001 (one-way ANOVA followed by Newman-Keuls multiple comparison test). Representative results from two or three independent experiments are shown (mean and s.d.).

**
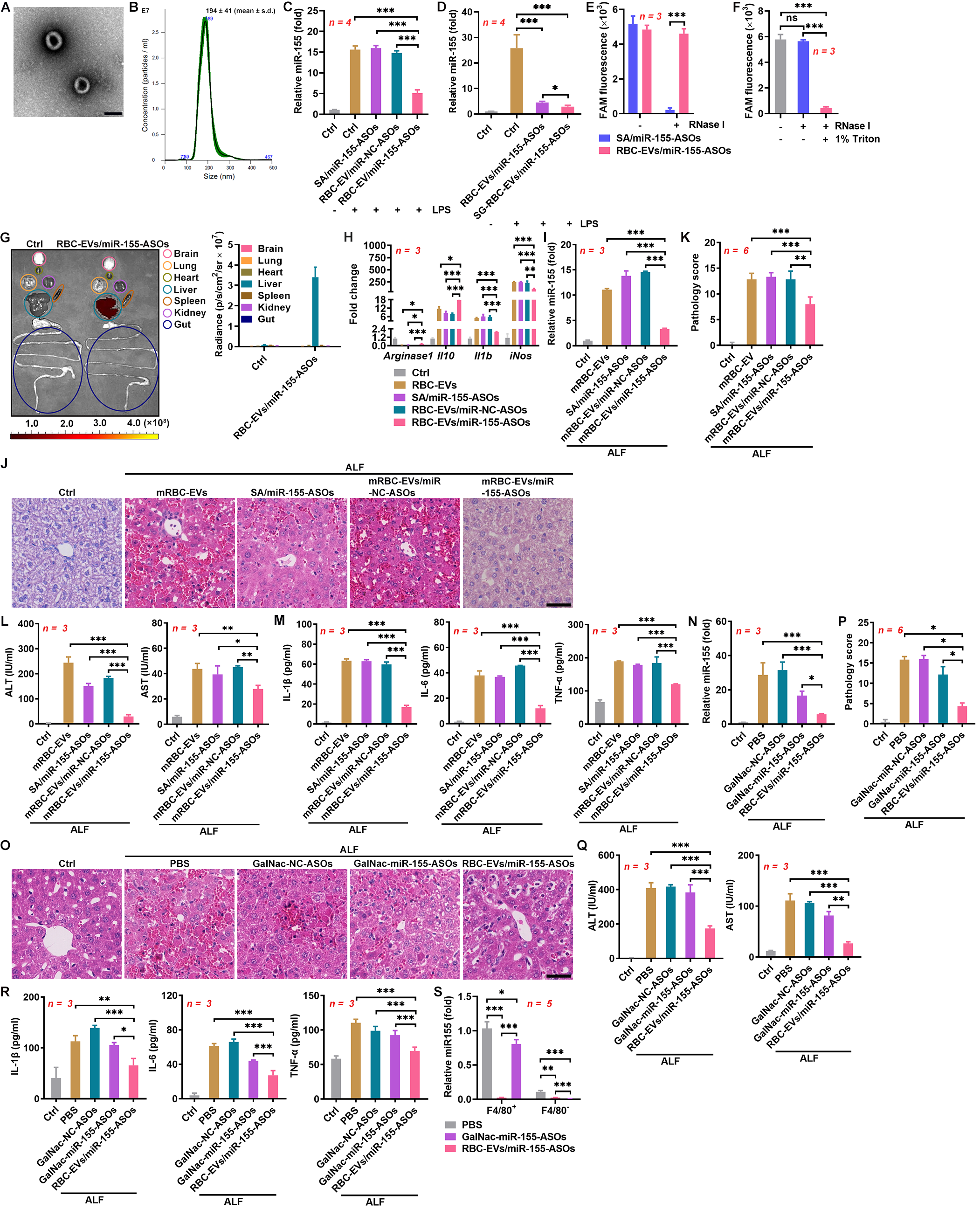
**

**Supplementary Figure 4: The RBC-EVs loaded with miR-155-ASOs protect against ALF**

(*A, B*) After loaded with miR-155-ASOs by electroporation, the morphology (*A*) and size distribution of the RBC-EVs (*B*) were detected by electron microscopy. Scale bar, 200 μm (*A*), and Nanoparticle Tracking Analysis (*B*). (*C, D*) After stimulation with 500 ng/ml LPS for 6 h, PMs were treated with SA/miR-155-ASOs (0.4 μg/ml miR-155-ASOs), 10 μg/ml RBC-EVs/miR-NC-ASOs or RBC-EVs/miR-155-ASOs (*C*), or 10 μg/ml RBC-EVs/miR-155-ASOs or SG-RBC-EVs/miR-155-ASOs (*D*) for 24 h. The miR-155 levels in the PMs were measured by real-time PCR. (*E, F*) FAM-labeled SA/miR-155-ASOs or 10 μg/ml RBC-EVs/miR-155-ASOs (*E*) or 10 μg/ml RBC-EVs/miR-155-ASOs disrupted by 1% Triton on ice for 1 h (*F*) were incubated with 100 units of RNase I for 4 h, and then the FAM fluorescence was measured. (*G*) Representative *ex vivo* imaging and quantification of organs from mice intravenously injected with 100 μg VivoTrack 680-labeled RBC-EVs/miR-155-ASOs (≈ 2.3 × 10^10^ particles) for 24 h. (*H*) Mice were intravenously injected with the SA/miR-155-ASOs (4 μg miR-155-ASOs), 100 μg RBC-EVs/miR-NC-ASOs or RBC-EVs/miR-155-ASOs on days -3, -2 and -1, and then ALF was induced in these mice by D-GalN/LPS on day 0. The mice were sacrificed, and the livers were isolated 24 h later. Levels of *Arginase1*, *Il10*, *Il1b* and *iNos* genes in liver macrophages were measured by real-time PCR. (*I-M*) Mice were intravenously injected with 100 μg RBC-EVs of C57BL/6J mice loaded with miR-NC-ASOs (mRBC-EVs/miR-NC-ASOs) or mRBC-EVs/miR-155-ASOs (≈ 2.3 × 10^10^ particles) on days -3, -2 and -1, and then ALF was induced in these mice by D-GalN/LPS on day 0. The mice were sacrificed, and the livers were isolated 24 h later. The miR-155 levels in the livers were measured by real-time PCR (*I*). Histopathological damage in the livers was detected by H&E staining. Scale bar, 40 μm (*J*), and the histopathological score was statistically analyzed (*K*). The levels of ALT and AST in sera were measured (*L*). The levels of IL-1β, IL-6 and TNF-α in sera were measured by ELISAs (*M*). (*N-S*) Mice were intravenously injected with 100 μg RBC-EVs/miR-155-ASOs or 4 μg GalNac-miR-155-ASOs on days -3, -2 and -1, and then ALF was induced in these mice by D-GalN/LPS on day 0. The mice were sacrificed, and the livers were isolated 24 h later. The miR-155 levels in the livers were measured by real-time PCR (*N*). Histopathological damage in the livers was detected by H&E staining. Scale bar, 40 μm (*O*), and the histopathological score was statistically analyzed (*P*). The levels of ALT and AST in sera were measured (*Q*). The levels of IL-1β, IL-6 and TNF-α in sera were measured by ELISAs (*R*). The miR-155 levels in F4/80^-^ and F4/80^+^ cells of the livers were measured by real-time PCR, and the reduction in miR-155 was calculated by dividing the relative miR-155 level in liver cells from mice without treatment by the relative miR-155 levels in liver cells from mice with the RBC-EVs/miR-155-ASOs treatment (*S*). ns, not significant; **P* < 0.05; ***P* < 0.01; ****P* < 0.001 (one-way ANOVA followed by Newman-Keuls multiple comparison test in *C*, *D*, *F*, *H*, *I*, *L*, *M*, *N*, *Q*, *R* and *S*; unpaired Student’s *t*-test in *E* or Kruskal-Wallis H test in *K* and *P*). Representative results from three independent experiments are shown (mean and s.d.).

**
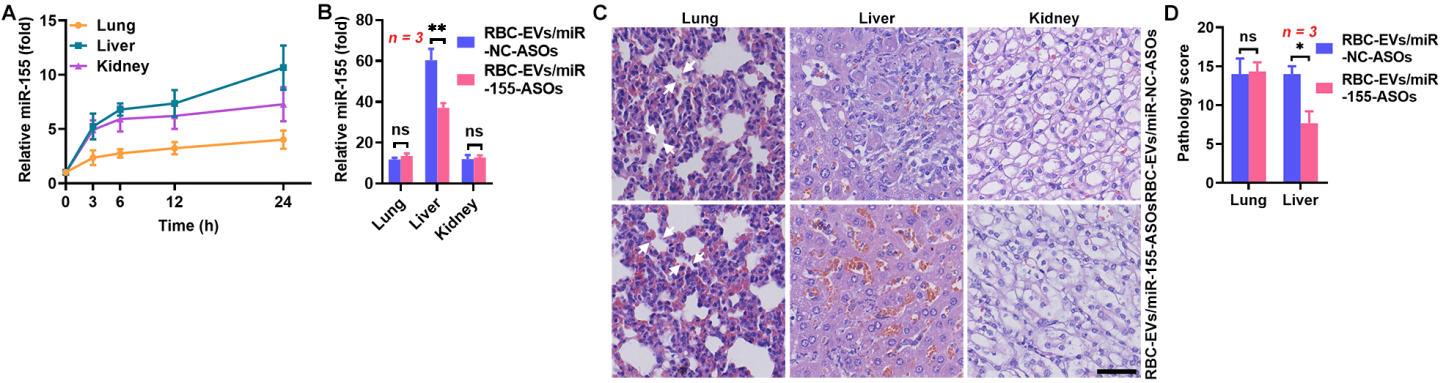
**

**Supplementary Figure 5: The RBC-EVs/miR-155-ASOs specifically protect sepsis in liver**

(*A*) After intraperitoneal injection with 500 μg/kg LPS for the indicated time, the miR-155 levels in the lungs, livers and kidneys were detected by real-time PCR. (*B-D*) Mice were intravenously injected with 100 μg RBC-EVs/miR-NC-ASOs or RBC-EVs/miR-155-ASOs (≈ 2.3 × 10^10^ particles) on days -3, -2 and -1, and then sepsis was induced in these mice by LPS on day 0. The mice were sacrificed, and the lungs, livers and kidneys were isolated 24 h later. The miR-155 levels in the lungs, livers and kidneys were measured by real-time PCR (*B*). Histopathological damage in the lungs, livers and kidneys was detected by H&E staining. Arrows indicate compressed alveoli. Scale bar, 40 μm (*C*), and the histopathological score was statistically analyzed (*D*). ns, not significant; **P* < 0.05; ***P* < 0.01 (unpaired Student’s *t*-test). Representative results from three independent experiments are shown (mean and s.d.).

**
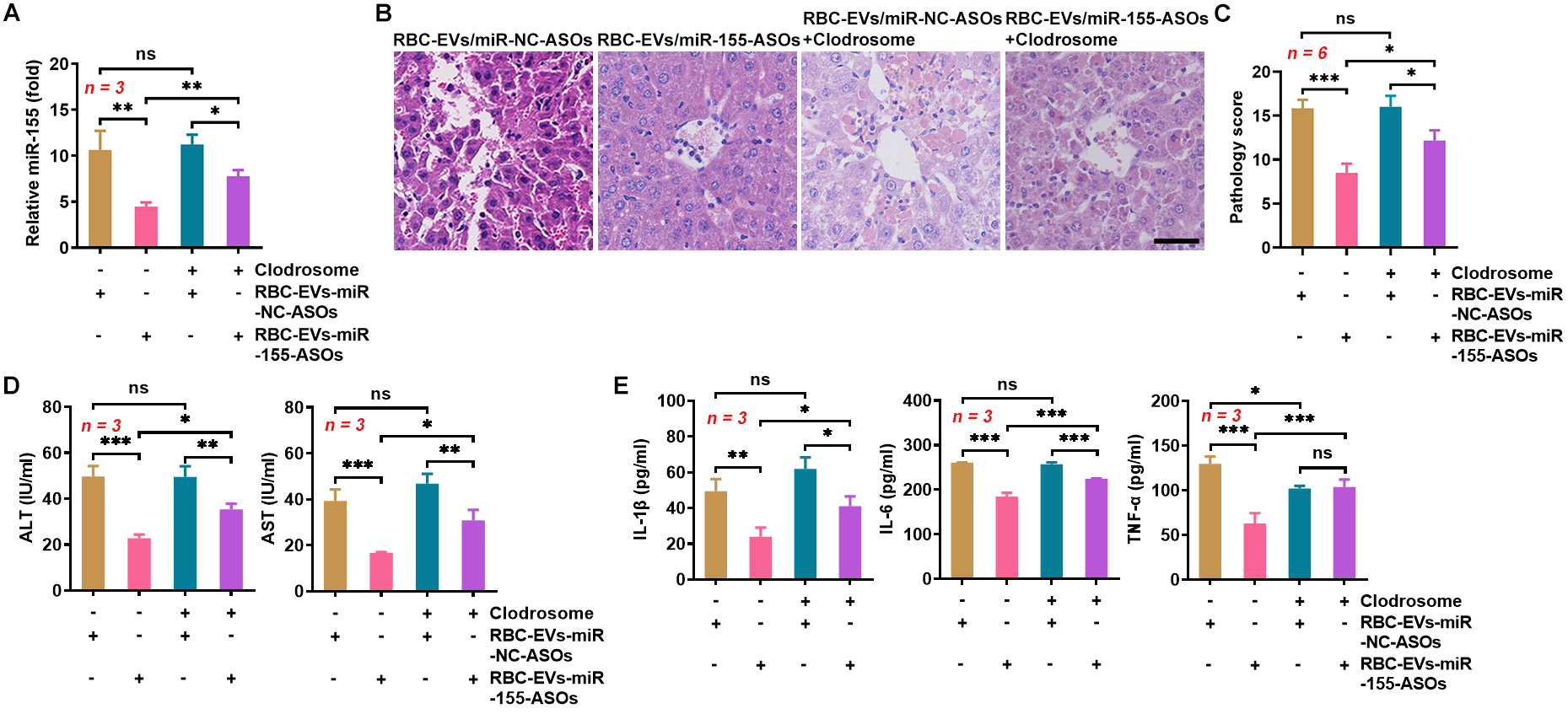
**

**Supplementary Figure 6: The RBC-EVs/miR-155-ASOs protect against ALF dependent of macrophage**

(*A-E*) Mice were intravenously injected with or without 50 μl Clodrosomes on days -4, -3, -2 and -1. These mice were also intravenously injected with 100 μg RBC-EVs/miR-NC-ASOs or RBC-EVs/miR-155-ASOs (≈ 2.3 × 10^10^ particles) on days -3, -2 and -1. Then, ALF was induced in these mice by D-GalN/LPS on day 0. The mice were sacrificed, and the livers were isolated 24 h later. The miR-155 levels in the livers were measured by real-time PCR (*A*). Histopathological damage in the livers was detected by H&E staining. Scale bar, 40 μm (*B*), and the histopathological score was statistically analyzed (*C*). The levels of ALT and AST in sera were measured (*D*). The levels of IL-1β, IL-6 and TNF-α in sera were measured by ELISAs (*E)*. ns, not significant; **P* < 0.05; ***P* < 0.01 and ****P* < 0.001 (one-way ANOVA followed by Newman-Keuls multiple comparison test in A, *D* and *E* or Kruskal-Wallis H test in *C*). Representative results from two independent experiments are shown (mean and s.d.).

**
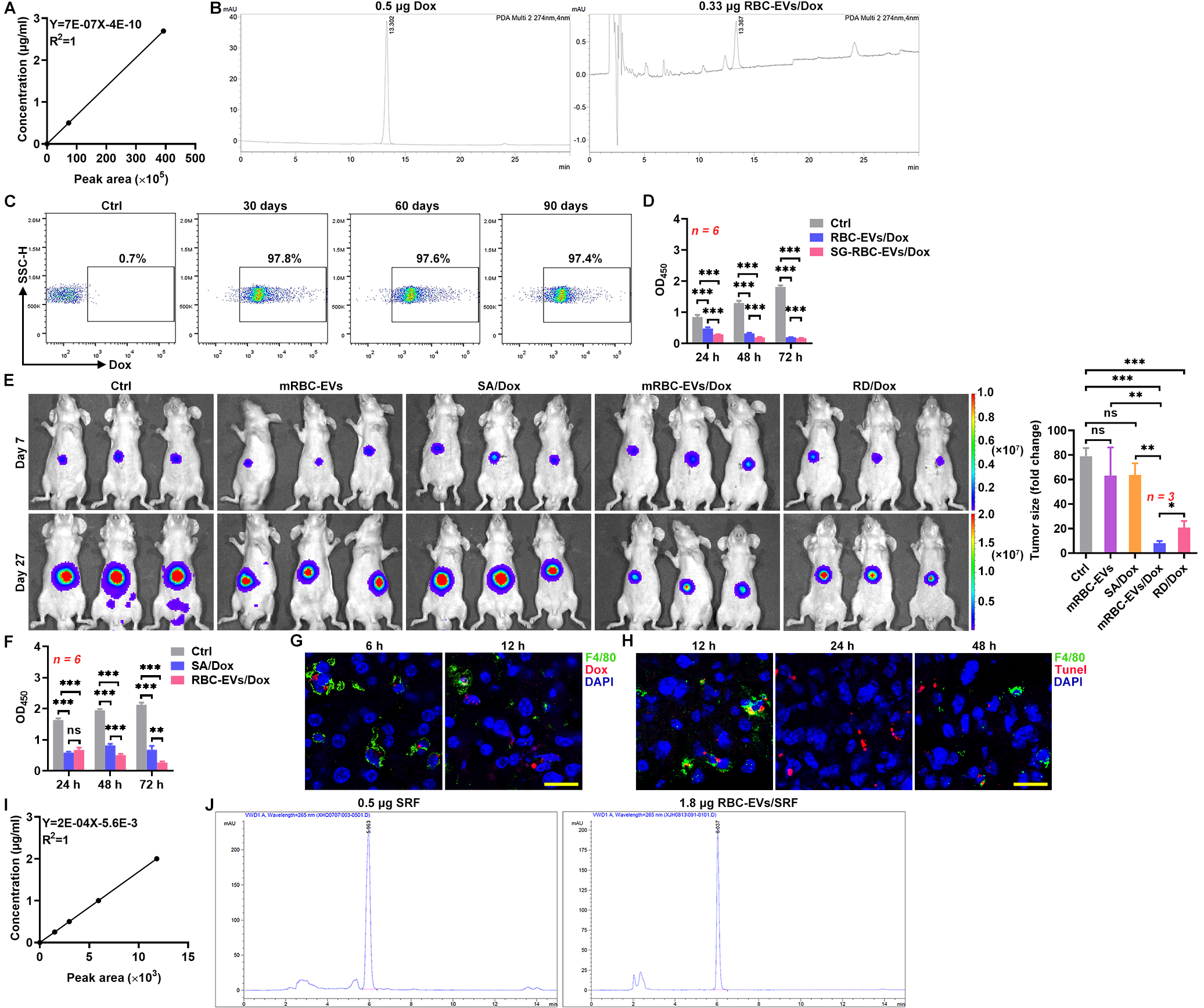
**

**Supplementary Figure 7:** **The** **RBC-EVs loaded with Dox or SRF inhibit orthotopic liver cancer growth**

1. Standard curve of the Dox concentration and the corresponding peak area obtained by HPLC analyses. (*B*) Representative results for 0.5 μg Dox standard and 0.33 μg RBC-EVs/Dox analyzed by HPLC. (*C*) After preservation at -80℃ for the indicated time length, Dox fluorescence in the RBC-EVs/Dox was detected by flow cytometry. (*D*) HCC-LM3 cells were treated with 10 μg/ml RBC-EVs/Dox or SG-RBC-EVs/Dox for the indicated time. Cell viability was measured by a CCK-8 assay. (*E*) HCC-LM3-Luci cells were orthotopically inoculated into mice on day 0. Then, the mice were intravenously injected with the 100 μg RBC-EVs (≈ 2.3 × 10^10^ particles) of C57BL/6J mice(mRBC-EVs), SA/Dox (3 μg Dox), 100 μg mRBC-EVs loaded with Dox (mRBC-EVs/Dox) and RD/Dox (5 mg/kg Dox) every 3 days starting on day 7. The tumor size was monitored by an IVIS system on days 7 and 27 (left). Tumor progression was evaluated through calculating the tumor size on day 27 divided by that on day 7 (right). (*F*) B16F10-Luci cells were treated with the SA/Dox (0.3 μg/ml Dox) or 10 μg/ml RBC-EVs/Dox for the indicated time. Cell viability was measured by a CCK-8 assay. (*G, H*) Mice were intravenously injected with 100 μg RBC-EVs/Dox (≈ 2.3 × 10^10^ particles) for the indicated time. Then, the distribution of Dox (*G*) and apoptosis (*H*) in F4/80^+^ and F4/80^-^ liver cells was detected by fluorescence microscopy. Scale bars, 20 µm. (*I*) Standard curve of the SRF concentration and the corresponding peak area obtained by HPLC analyses. (*J*) Representative results for 0.5 μg SRF standard and 1.8 μg RBC-EVs/SRF analyzed by HPLC. ns, not significant; ***P* < 0.01 and ****P* < 0.001 (one-way ANOVA followed by Newman-Keuls multiple comparison test). Representative results from three independent experiments are shown.

**
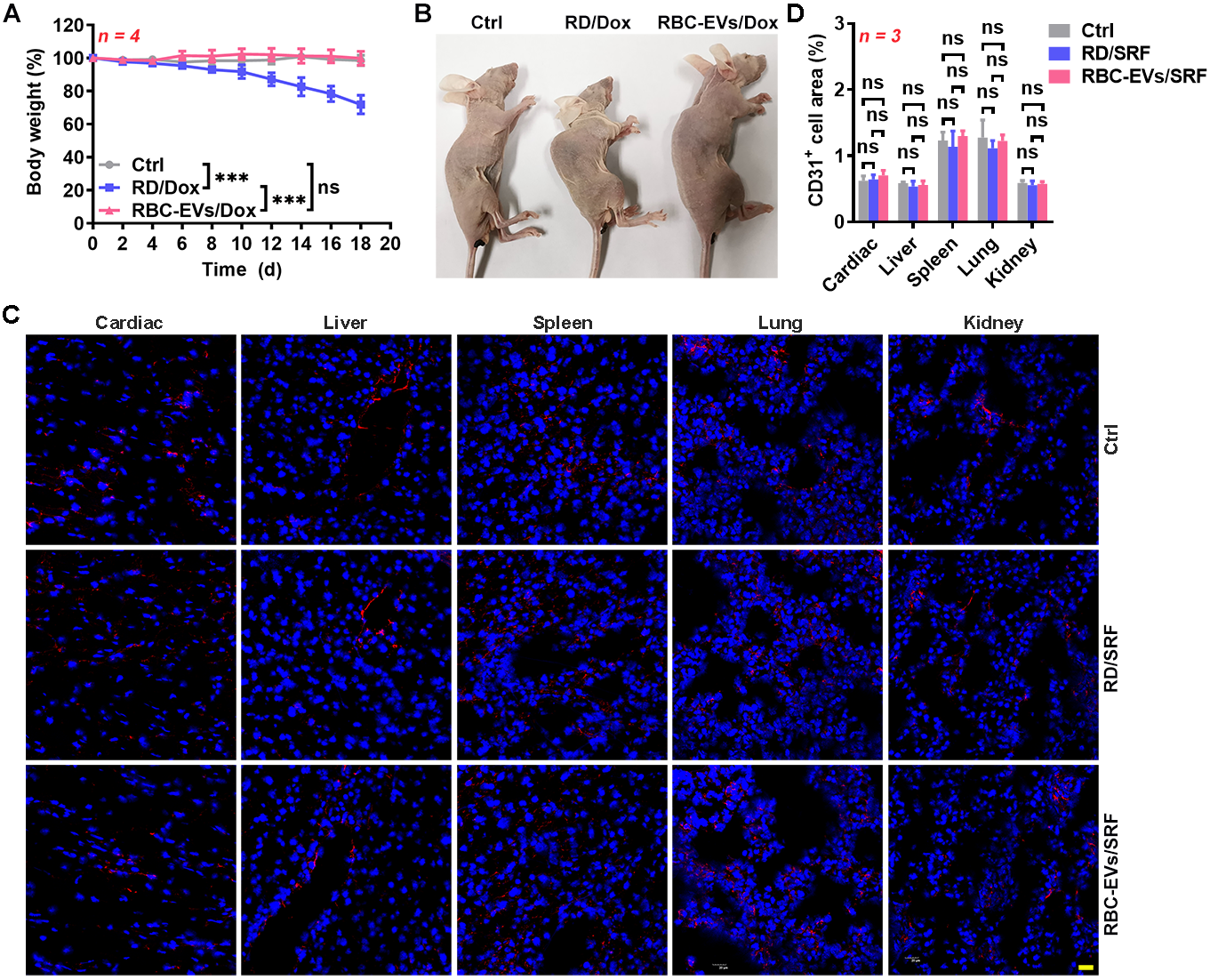
**

**Supplementary Figure 8:** **Toxicity evaluation of the drug-loaded RBC-EVs**

(*A, B*) Mice were intravenously injected with the RD/Dox (5 mg/kg Dox) or 100 μg RBC-EVs/Dox (≈ 2.3 × 10^10^ particles) every 3 days starting on day 0. Body weight was measured every other day (*A*), and the appearance of mice on day 18 is shown (*B*). (*C*) Mice were intravenously injected with the RD/SRF (30 mg/kg SRF) or 100 μg RBC-EVs/SRF (≈ 2.3 × 10^10^ particles) every 3 days starting on day 0. On day 18, the mice were sacrificed, and angiogenesis in the indicated organs was detected by CD31 staining. Scale bars, 20 µm. (*D*) The CD31^+^ cell area in (*C*) was statistically analyzed. ns, not significant, ****P* < 0.001 (two-way ANOVA in *A* or one way ANOVA in *D* followed by Newman-Keuls multiple comparison test). Representative results from three independent experiments are shown (mean and s.d.).

**Supplementary Table 1: The characteristics of tumor patients**

| **Patient number** | **Gender** | **Age** | **Diagnose** | **Clinical stage** |
| --- | --- | --- | --- | --- |
| 1 | Male | 54 | Lung adenocarcinoma | Ⅲa |
| 2 | Male | 48 | Lung squamous carcinoma | Ⅱa |
| 3 | Male | 60 | Lung adenocarcinoma | Ⅰb |
| 4 | Male | 67 | Gastric adenocarcinoma | Ⅰb |
| 5 | Female | 72 | Gastric adenocarcinoma | Ⅱa |
| 6 | Male | 55 | Gastric adenocarcinoma | Ⅲa |
| 7 | Male | 73 | Hepatocellular carcinoma | Ⅲa |
| 8 | Female | 64 | Hepatocellular carcinoma | Ⅱa |
| 9 | Male | 66 | Hepatocellular carcinoma | Ⅲa |

**Supplementary Table 2: The antibodies for** **immunofluorescence, immunohistochemistry, immunoblotting and flow cytometry**

| **Antibodies** | **Source** | **Identifier** | **Dilution ratio** |
| --- | --- | --- | --- |
| **αSMA** | Cell Signaling Technology | 19245 | 1: 200 |
| **CD31** | Abcam | ab56299 | 1: 200 |
| **Albumin** | Abcam | ab207327 | 1: 200 |
| **F4/80** | Abcam | ab6640 | 1: 200 |
| **CD68** | Abcam | ab213363 | 1: 200 |
| **Hemoglobin A1** | ABclonal | A7322 | 1: 1000 |
| **ARF6** | ABclonal | A0988 | 1: 1000 |
| **Alix** | ABclonal | A2215 | 1: 1000 |
| **Tsg101** | ABclonal | A2216 | 1: 1000 |
| **HSP70** | BD biosciences | 610607 | 1: 1000 |
| **C1q** | Abcam | ab71940 | 1: 1000 |
| **GAPDH** | ABclonal | AC033 | 1: 5000 |
| **β-actin** | Huabio | ET1702-52 | 1: 5000 |
| **goat anti-rabbit (HRP)** | Abcam | ab6721 | 1: 1000 |
| **goat anti-rabbit (HRP)** | MultiScience | GAR007 | 1: 5000 |
| **goat anti-rat (HRP)** | Abcam | ab97057 | 1: 1000 |
| **goat anti-mouse (HRP)** | MultiScience | GAM0072 | 1: 5000 |
| **goat anti-rat (Alexa Fluor 488)** | Abcam | ab150157 | 1: 200 |
| **goat anti-rat (Alexa Fluor 594)** | Abcam | ab150160 | 1: 200 |
| **goat anti-rabbit (Alexa Fluor 488)** | Abcam | ab150077 | 1: 200 |
| **goat anti-rabbit (Alexa Fluor 594)** | Abcam | ab150080 | 1: 200 |
| **FITC anti-mouse CD47** | Invitrogen | 11-0471-82 | 1: 400 |
| **APC anti-human CD47** | Invitrogen | 17-0479-42 | 1: 400 |
| **FITC anti-mouse F4/80** | Invitrogen | 11-4801-82 | 1: 400 |
| **APC anti-mouse F4/80** | Invitrogen | 17-4801-82 | 1: 400 |
| **PE anti-mouse CD11b** | Invitrogen | 12-0112-82 | 1: 400 |
| **FITC anti-mouse CD11b** | Invitrogen | 11-0112-82 | 1: 400 |
| **FITC anti-human CD11b** | Invitrogen | MA5-16529 | 1: 400 |
| **PE anti-human CD68** | Invitrogen | 12-0689-42 | 1: 400 |
| **PE anti-mouse CD68** | Invitrogen | MA5-16678 | 1: 400 |
| **APC anti-mouse CD206** | Invitrogen | 17-2061-82 | 1: 400 |
| **APC anti-mouse SIRPα** | Invitrogen | 17-1721-82 | 1: 400 |

**Supplementary Table 3: Sequences used in this article**

| **Sequence for anti-miR-155 ASOs** | |
| --- | --- |
| **miR-155 ASOs** | 5’-ACCCCUAUCACAAUUAGCAUUAA-3’ |
| **miR-NC-ASOs** | 5’-CAGUACUUUUGUGUAGUACAA-3’ |
| **Primers for real-time PCR** | |
| ***mActb* F** | 5’-GGCTGTATTCCCCTCCATCG-3’ |
| ***mActb* R** | 5’-CCAGTTGGTAACAATGCCATGT-3’ |
| ***mmiR-155* F** | 5’-CGGCGGTTAATGCTAATTGTGAT-3’ |
| ***mmiR-155* R** | 5’-GTGCAGGGTCCGAGGT-3’ |
| ***mU6* F** | 5’-CAGCACATATACTAAAATTGGAACG-3’ |
| ***mU6* R** | 5’-ACGAATTTGCGTGTCATCC-3’ |
| ***m******SIRPα* F** | 5’-AGTCACGGGGAAAGAACTGAA-3’ |
| ***mSIRPα* R** | 5’-CGGCTTTGCCTACTCCTCTG-3’ |
| ***mC1qa* F** | 5’-AAAGGCAATCCAGGCAATATCA-3’ |
| ***m******C1qa* R** | 5’-TGGTTCTGGTATGGACTCTCC-3’ |
| ***mIL-1β* F** | 5’-GCAACTGTTCCTGAACTCAACT-3’ |
| ***mIL-1β* R** | 5’-ATCTTTTGGGGTCCGTCAACT-3’ |
| ***miNOS* F** | 5’-GTTCTCAGCCCAACAATACAAGA-3’ |
| ***miNOS* R** | 5’-GTGGACGGGTCGATGTCAC-3’ |
| ***mArginasel* F** | 5’-CTCCAAGCCAAAGTCCTTAGAG-3’ |
| ***mArginasel* R** | 5’-AGGAGCTGTCATTAGGGACATC-3’ |
| ***mIL-10* F** | 5’-GCTCTTACTGACTGGCATGAG-3’ |
| ***mIL-10* R** | 5’-CGCAGCTCTAGGAGCATGTG-3’ |
| **Sequences for siRNA** | |
| ***mC1qa siRNA* F** | 5’-UGAAAGGCAAUCCAGGCAAUATT-3’ |
| ***mC1qa siRNA* R** | 5’-UAUUGCCUGGAUUGCCUUUCATT-3’ |
| **NC *siRNA* F** | 5’-UUCUCCGAACGUGUCACGU-3’ |
| **NC *siRNA* R** | 5’-ACGUGACACGUUCGGAGAA-3’ |
